# Applications of Medical Mediation: A Systematic Review of Its Role in Healthcare Dispute Resolution and Bioethical Decision-Making

**DOI:** 10.3390/healthcare13243235

**Published:** 2025-12-10

**Authors:** Olympia Lioupi, Polychronis Kostoulas, Konstadina Griva, Charalambos Billinis, Costas Tsiamis

**Affiliations:** 1 Faculty of Public and One Health, School of Health Sciences, University of Thessaly, 43100 Karditsa, Greece; pkost@uth.gr (P.K.); ktsiamis@uth.gr (C.T.); 2 Lee Kong Chian School of Medicine, Nanyang Technological University, Singapore 639798, Singapore; konstadina.griva@ntu.edu.sg; 3 Faculty of Veterinary Medicine, School of Health Sciences, University of Thessaly, 43100 Karditsa, Greece; billinis@uth.gr

**Keywords:** medical mediation, bioethics mediation, healthcare dispute resolution, clinical ethics, patient–provider conflict, systematic review

## Abstract

**Background**: Medical mediation offers a patient-centered, collaborative alternative to traditional resolution methods for healthcare conflicts that is gaining international traction in an increasingly complex environment of advancing technology and diverse patient populations. This systematic review aims to synthesize the literature on medical mediation and analyze its clinical applications, conflict typologies, involved actors, mediation methodologies, legal frameworks, and theoretical underpinnings. **Methods**: A systematic search was conducted in PubMed and Scopus for English-language articles published between 1984 and 2025. **Results**: Of 656 initial records, 152 studies met the inclusion criteria and were categorized across six domains: clinical context, actors involved, conflict type, mediation framework, legal/policy structure, and theoretical foundations. Most studies originated from high-income countries, particularly the U.S. and U.K., with notable expansion after 2010. Medical mediation was most frequently applied in bedside care, end-of-life decision-making, and managed-care disputes. While ethics consultants were the primary mediators, increasing involvement of trained clinicians and institutional actors was also observed. Most studies emphasized generic bioethical mediation frameworks, with some focused on formalized models and training. Legal frameworks varied, and an increasing number of countries have been adopting institutional or national programs to support mediation. **Conclusions**: Medical mediation is an efficient tool for resolving complex clinical conflicts, enhancing communication, and preserving therapeutic relationships. Its institutionalization, through law and training, is key to the promotion of justice, transparency, and ethical integrity in modern healthcare systems.

## 1. Introduction

Conflicts in healthcare settings, whether due to medical errors, disputes over end-of-life care, or cultural and religious values, are inevitable in an era of advancing medical technology and diverse patient populations. Traditional approaches to resolving these conflicts, such as litigation or ethics consultations, often prioritize negative outcomes or top-down decision-making, thereby compromising trust between patients, families, and providers [1,2]. In contrast, medical mediation has emerged as a collaborative, patient-centered alternative that utilizes neutral third-party facilitation to enhance dialog, mutual understanding, and consensus [3]. It shifts the focus from “winning” to shared problem-solving and, in this way, addresses immediate differences and preserves therapeutic relationships and institutional integrity.

Medical mediation as a standardized practice began to take shape in the United States in the late 1970s and early 1980s, with initiatives focused on addressing bioethical conflicts, particularly those relate to end-of-life decision-making. Nancy Dubler introduced the “bioethics mediation” model in the 1980s and 1990s, highlighting the importance of a neutral third party in resolving complex clinical disputes [4]. In the 2000s, the practice was further strengthened by empirical research that highlighted the effectiveness of mediation in reducing litigation and improving communication between patients and medical teams. For example, studies highlighted mediation as a tool for resolving conflicts in medical malpractice cases [2] and examined its effectiveness in hospitals [1]. The spread of medical mediation accelerated after 2010, with countries developing specific protocols, such as the United Kingdom’s protocol for conflicts in pediatric care [5] and others, like China’s incorporation of mediation into medical malpractice laws [6]. As the pressure for viable alternatives to litigation, the need for enhanced transparency, and better management of ethically complex scenarios has increased [5], medical mediation has been spreading. Thus, over the past four decades, the adoption of medical mediation has expanded worldwide. However, despite its growing prominence there is limited synthesis of its applications, methodologies, and effectiveness across different healthcare systems.

The objective of this systematic review is to address this gap through the collection and analysis of published studies on medical mediation in order to (1) map the clinical settings where it is applied; (2) identify the types of conflicts and (3) actors involved; (4) examine the mediation methodologies and legal contexts; and (5) explore the theoretical foundations and normative dimensions that frame its implementation across healthcare systems. A comprehensive synthesis of the empirical, legal, and ethical dimensions of medical mediation can inform both policy and practice in an increasingly complex healthcare landscape.

## 2. Methods

### 2.1. Search Strategy and Eligibility Criteria

A systematic literature search was conducted in PubMed and Scopus to identify studies related to medical mediation. The search was restricted to studies published in the last 40 years and covered the period from January 1984 to November 2025. The last search was performed on 20 November 2025. The following Boolean search string was applied in each database: (“medical mediation” OR “bioethics mediation” OR “bioethical mediation” OR “healthcare mediation” OR “clinical ethics mediation”). Although the primary search strategy targeted English-language publications, studies in other languages were also included when full texts or high-quality translations were available through the databases or reference lists. Initially, a total of 656 records was retrieved, which, after checking and removing duplicate entries, resulted in a final dataset of 639 unique studies for further screening and analysis.

The eligibility screening was conducted by two reviewers (OL and PK), who screened all titles and abstracts against the predefined criteria. Disagreements were resolved through discussion and consensus. The inclusion criteria included the following: (1) articles explicitly referencing medical mediation, bioethics mediation, or healthcare conflict resolution; (2) articles describing mediation practices, legal frameworks, or ethical consultations; and (3) articles describing empirical, conceptual, or case-based focus on the application of medical mediation. Articles were excluded if they were not related to the use of medical mediation in healthcare and/or if they were without substantive reference to medical mediation. A total of 174 reports were sought for retrieval, of which 6 could not be retrieved despite multiple attempts. Full-text assessment was conducted for 168 reports, again by two independent reviewers. Sixteen reports were excluded because they did not provide substantive discussion of medical mediation (e.g., ethics consultations without mediation, or general conflict communication without a mediation component). A total of 152 studies met the eligibility criteria and were included in the final synthesis.

This systematic review was conducted in accordance with the PRISMA 2020 guidelines (Supplementary Table S1) [7]. No protocol was registered for this review. Because this work synthesizes conceptual, ethical, legal, and policy-oriented literature, including opinion papers, theoretical analyses, and descriptive frameworks, it does not meet the eligibility criteria for registries such as PROSPERO, which focus on systematic reviews of clinical or quantitative health outcomes. Therefore, protocol registration was not applicable to the present review.

### 2.2. Risk of Bias and Quality Appraisal

The methodological quality and potential risk of bias of all included studies were assessed using the appropriate critical appraisal tools from the Joanna Briggs Institute—JBI [8]. Given the heterogeneity of study types, which ranged from qualitative research and case studies to text and opinion papers, we applied the following tools: (a) the JBI Critical Appraisal Checklist for Qualitative Research, (b) the JBI Checklist for Case Reports, and (c) the JBI Checklist for Text and Opinion Papers. Two reviewers (OL and PK) independently assessed the studies, and disagreements were resolved through discussion. Each study received an overall judgment of low, moderate, or high concern regarding methodological quality. The full set of JBI appraisal results for all 152 studies is presented in Supplementary Table S2.

### 2.3. Selection and Categorization

The articles that met the inclusion criteria were selected for full-text review and were subsequently analyzed in depth. For each manuscript we assessed its content in relation to five domains:

1. Clinical context of mediation. In this category, we classified the articles based on where the mediation takes place or which aspect of clinical practice was addressed. The subcategories were as follows: (1) end-of-life care and medical futility; (2) pediatrics and neonatal intensive care units or intensive care units (NICUs or ICUs, respectively); (3) general—bedside/clinical practice; (4) managed care/administrative and legal; and (5) long-term care or rehabilitation.
2. Actors involved in mediation. Articles were classified based on the types of actors involved. The categories were as follows: (1) ethics consultants or committees acting as mediators; (2) physicians, nurses, and health professionals trained in mediation; and (3) institutional or third-party mediators.
3. Types of conflict in mediation. Articles were classified into the following categories: (1) medical errors, adverse events and malpractice; (2) religious, cultural, and value conflicts; (3) resource allocation and priority setting; and finally, (4) patient—provider relationship conflicts.
4. Mediation methodologies and frameworks. This category classified articles based on the types, models, or methods of mediation used. It had the following categories: (1) named or formal mediation frameworks; (2) generic or conceptual bioethics mediation; (3) mediation training and education; and (4) proactive or preventive ethics/early warning models.
5. Legal and policy frameworks. This domain categorized articles that discussed either (1) national or institutional mediation programs or (2) the intersection of ethics and law.
6. Theoretical, philosophical, or normative analyses. This last domain included articles that addressed theoretical–philosophical aspects of medical mediation (also in relation to alternatives) or normative structures underlying medical mediation.

**Figure 1:**
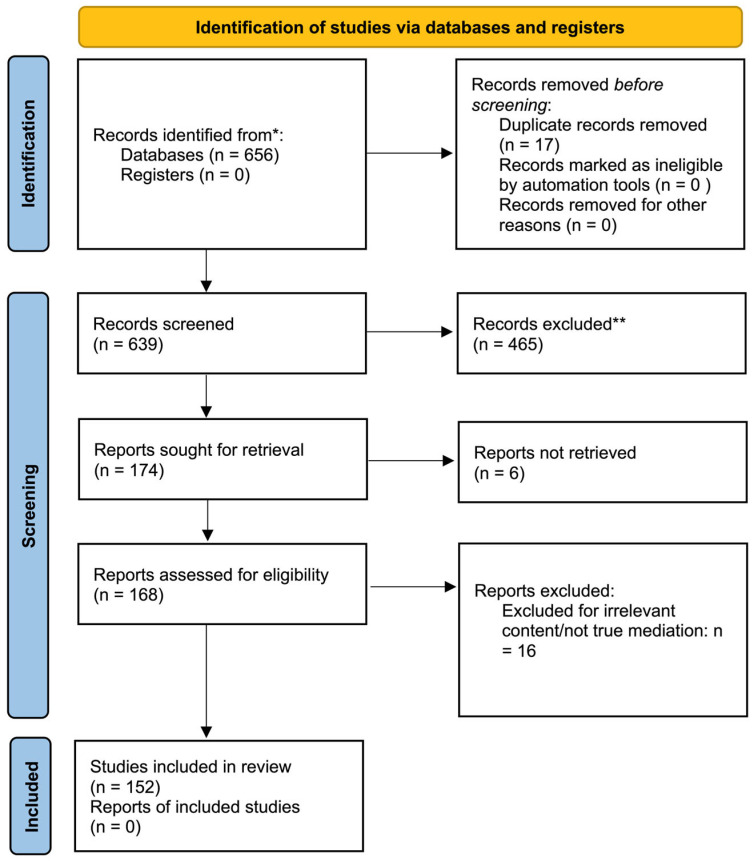
PRISMA 2020 flow diagram of study selection. * Records identified from database searches (PubMed and Scopus) after removal of automated duplicates. ** Records excluded during title and abstract screening because they did not refer to medical mediation or were clearly irrelevant to the review topic.

An article, based on a review of its full content, could be listed into any of these categories but not necessarily in all of them. Of the 639 screened abstracts, 152 articles that met the inclusion criteria were selected for full-text review and thematic categorization. The study selection process is illustrated in the PRISMA flow diagram (Figure 1).

## 3. Findings

### 3.1. Included Studies

**Figure 2:**
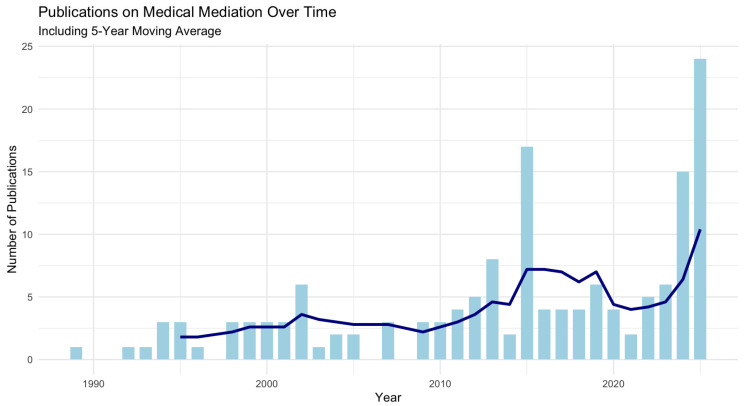
Temporal trends in publications on medical mediation (1984–2025). Light blue bars indicate annual publication counts, while the dark blue line represents a 5-year moving average to illustrate overall trends.

A total of 152 articles were included following the full-text screening and evaluation (Figure 1); the articles ranged over a 40-year period, from 1984 to 2025, with a notable increase in the number of publications over the past two decades. Number of publications per year is shown in Figure 2. The majority of studies originated from high-income countries such as the United States, the United Kingdom, and Canada. However, contributions from Asian, European, and Latin American countries were also identified. Across all articles, six key domains were used to categorize the framing of medical mediation for each manuscript.

### 3.2. Risk-of-Bias Assessment

The overall methodological quality of the included studies varied considerably, reflecting the diversity of study designs. JBI appraisal indicated that most qualitative and case-based studies exhibited low to moderate concern, primarily related to reflexivity, clarity of context, and reporting transparency. Only a small subset of studies presented high concern, typically due to insufficient methodological detail.

### 3.3. Study Categorization

**Figure 3:**
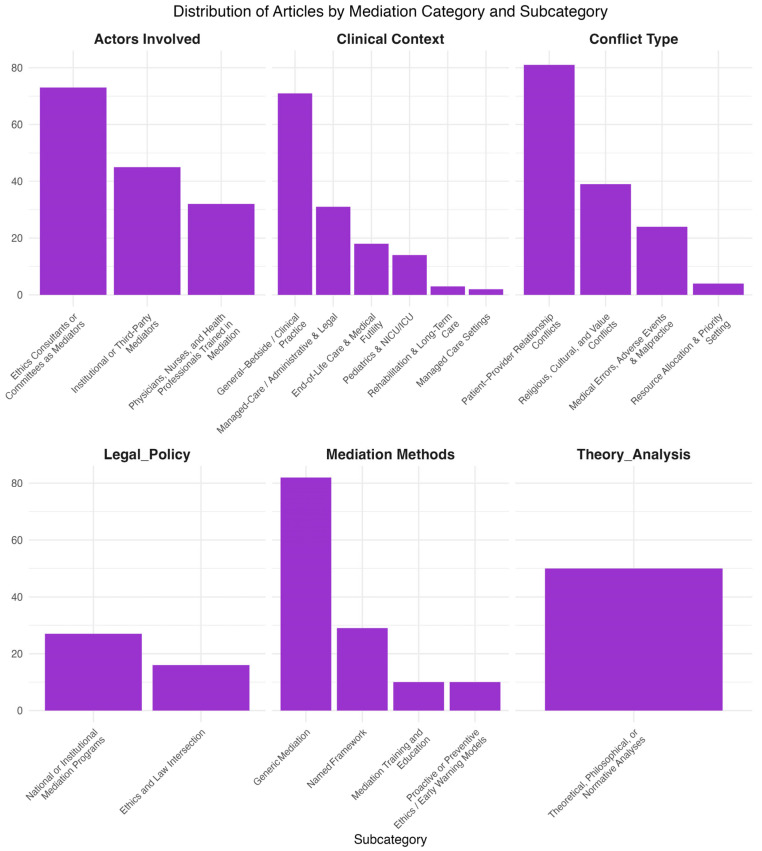
Distribution of articles by (1) actors involved, (2) clinical context, (3) conflict type, (4) legal and policy framework, (5) mediation methods, and (6) theoretical or normative analysis. Within each domain, articles were categorized into subdomains reflecting their primary focus. Most studies addressed patient–provider conflicts, ethics consultants as mediators, and general bedside or clinical practice contexts.

The distribution of number of articles by subcategory in each domain is shown in Figure 3.

In the clinical-context domain, the majority of the articles discussed medical mediation in the case of general bedside—clinical practice, followed by managed the care/administrative and legal general contexts. The most common single context was end-of-life care and medical futility, addressed in approximately 18 out of 152 articles, followed by the use of mediation in pediatrics and ICU settings.

For the actors involved domain, ethics consultants or committees acting as mediators were the most prominent, appearing in 73 out of 152 studies, while the other two categories, (a) physicians, nurses, and health professionals trained in mediation and (b) institutional or third-party mediators, appeared in comparable numbers of publications.

Of the four major subcategories of type of conflict, the vast majority of articles addressed patient—provider relationship conflicts followed by religious, cultural, and value conflicts, while the most common context-specific subcategory was the one related to the use of medical mediation as a tool to resolve medical errors, adverse events, and malpractice cases.

For the legal policy domain, most articles dealt with national or institutional mediation programs compared to those on the intersection of ethics and law. In terms of mediation methodologies and frameworks, the vast majority of articles referenced different types of bioethics mediation models, with most of them being generic discussions, followed by articles dealing with named mediation modeling frameworks. Finaly, 50 out of 152 articles contributed to the theoretical foundations of medical mediation. These domains also included critiques of the “consultation” model in clinical ethics, arguments for mediation as a non-adversarial alternative, and discussions of philosophical counseling as a parallel or complementary process.

Many studies spanned multiple domains. For instance, a significant proportion of articles that focused on end-of-life care also involved ethics consultants and bioethics mediation frameworks. Others combined empirical accounts of institutional training with discussions of national policy.

## 4. Discussion

This systematic review provides a comprehensive synthesis of the literature on medical mediation, mapping its clinical applications, the different types of actors involved, the various types of methods implemented in the different types of conflict, and the existing legal frameworks across diverse healthcare settings and jurisdictions. The evolution of medical mediation over the past decades has been followed by an analogous rise in published research. From a handful of articles in the 1980s/1990s [9,10], we have observed a steady increase in the number of studies on medical mediation, especially after 2000, with a peak after 2010 (Figure 2). One of the potential main drivers for this increase is the rise in the demand and supply of clinical ethics services, especially in malpractice reform [11]. Starting from the United States and followed by the United Kingdom, where medical mediation first gained momentum, with early implementations linked to tort reform and initiatives aimed at reducing litigation and improving dispute resolution in healthcare [12], it further expanded with the institutionalization of mediation through national legal frameworks in several countries, such as South Korea [13] and China [6]. This expansion also led to a shift from early papers that were mostly descriptive and/or theoretical [14] towards recent work, which is empirical [15], intervention-based [16], and policy-oriented.

A notable pattern within the literature is the predominance of Anglo-American scholarship, particularly the conceptual and clinical ethics mediation models. This pattern reflects the historical emergence of medical mediation within the U.S. tort-reform movement and the early institutionalization of clinical ethics services, rather than a methodological limitation of this review. Recent analyses of mediation in healthcare similarly observed that most structured mediation frameworks for reforms originated in U.S. and U.K. systems before diffusing globally [17]. Importantly, the global evidence base recovered in this synthesis demonstrates that medical mediation has also evolved in diverse legal and cultural environments. Studies from China [6], Taiwan [6], Japan [18], Italy [19], Spain [20], Poland [21], France [22], Greece [23,24], Panama [25], and Brazil [26] illustrate alternative mediation models, statutory mechanisms, and culturally embedded approaches to conflict resolution. It is also important to acknowledge that mediation has deep historical roots in Eastern legal and philosophical traditions, particularly in China and other East Asian societies, where harmony-oriented, conciliatory approaches to conflict predate modern ADR frameworks. Recognizing these jurisdictional variations helps situate the Anglo-American tradition within a broader international landscape and underscores that medical mediation is neither uniform nor culturally neutral. The expansion of medical mediation beyond Anglo-American systems reflects a wider European and Mediterranean movement toward structured dispute resolution in healthcare. Importantly, mediation practices are shaped not only by ethics consultation models but also by diverse legal cultures, family structures, and communication norms.

For the clinical context domain, most of the articles (71 out of 152) fall within the general bedside—clinical practice category, which was most commonly framed as a function of ethics consultation, especially in the case of moral uncertainty, patient–provider disputes, and cultural or religious value conflicts. Several of the studies conducted advocated for mediation as a replacement for or enhancement of traditional ethics consultations, with repeated emphasis on communication, empathy, and collaborative resolution [2,27,28]. Furthermore, several studies also highlighted the dynamic and continuously evolving role of the clinical ethicist as a mediator, especially within high-conflict care environments [29,30]. The second most common category was that of managed care, administrative and legal (31 out of 152), which explored the use of mediation in resolving conflicts associated with insurance coverage, billing, and resource denial. In addition, several studies focused on institutional trust [31], apology frameworks [32], and programs aiming at resolution through communication [5,11]. End-of-life care and medical futility was third and the most common single issue (18 out of 152 articles) addressed across the literature. Mediation is a key tool frequently employed to de-escalate ICU disputes, reach consensus (e.g., on withdrawing treatment), and manage surrogate decision-making conflicts [33,34,35]. Pediatric and ICU settings mainly addressed parent–provider conflict escalation [36], especially in end-of-life scenarios in pediatric oncology and neonatal care, and also pointed out the key role of structured mediation frameworks and training programs like the UK Medical Mediation Foundation [37]. Finally, in long-term care and rehabilitation settings, mediation plays a critical role in resolving care planning and value conflicts [38], especially in geriatric settings [39] and nursing homes [40]. This distribution of actors also suggests that mediation often emerges in cases where traditional decision-making processes prove insufficient for resolving conflict, as is the case when institutional authority, professional hierarchy, and moral uncertainty intersect.

From the actors involved, the category of ethics consultants/committees was the most frequently addressed, with some studies pointing out facilitator–mediator hybrids [3] and others framing mediation within the role of ethics committees [41]. Physicians, nurses, and physician assistants were the second category depicted as acquiring mediation skills, especially those serving in intensive care [42], palliative [43], and pediatric settings [44]. Finally, an equal volume of research articles addressed the role of institutional and third-parties as mediators, like court-appointed panels and insurance-based mediation schemes, which function outside the traditional clinical ethics infrastructure and offer formalized processes [17,45]. This distribution of actors also suggests that mediation often emerges where institutional authority, professional hierarchy, and moral uncertainty intersect, that is, in settings where traditional decision-making processes prove insufficient for resolving conflict.

Among the types of conflict, the most common category was that of the patient–provider relationship, with articles dealing mainly with the issues of trust erosion [46], handling of difficult patients [47], and conflicts arising from delayed care, as in the case of HIV [48]. The next reason for conflict was due to religious, cultural, and value conflicts. Studies found that religious disputes could be approached through mediation [49], as could other multicultural bedside dilemmas and other crucial moral issues such as stem-cell ethics. Medical errors and malpractice was another important category, with several articles emphasizing the role of communication and resolution mediation programs (CRPs) as a tool for structured dialog and early disclosure [5,18]. An additional important aspect of medical mediation relates with how apologies can rebuild trust and reduce the risk of long-term conflict [18,32]. Further, the existence of mediation committees in national contexts, such as Taiwan [6] and China [6], was also discussed as part of the formal mechanisms to resolve disputes. Finally, few articles discussed conflicts due to resource allocation and priority setting. These investigated the use of mediation in the case of insurance denials, ICU triage decisions, and hospital-based appeals processes, where mediation was proposed as a means to enhance transparency and legitimacy [50,51]. Across jurisdictions, these conflict categories reveal a recurring pattern of mediation most frequently being mobilized when disputes involve relational breakdown (trust, communication), perceived unfairness (resource allocation, insurance denial), or moral pluralism (religious and cultural values). European studies, in particular, emphasize mediation’s role in bridging communication gaps in publicly funded systems where shared decision-making and family involvement are culturally normative. A recent review [17] highlights how communication breakdown and relational expectations between patients, families, and clinicians make mediation especially valuable in European healthcare contexts.

In terms of the mediation methodologies and frameworks used in mediation, the vast majority of the published literature falls within the category of generic—conceptual mediation and emphasizes the centrality of structured dialogue, moral negotiation, and ethical facilitation. Moreover, it advocates for mediation as a more dynamic and participatory alternative [2,52]. Mediation better addresses moral uncertainty and facilitates closure in high-conflict cases. Further, several articles discussed named or formal mediation frameworks, like the Dubler–Fisher bioethics mediation model, which provides a structured approach to mediation based on narrative engagement and consensus-building [53,54]; the commitment model, which emphasizes collaborative deliberation and shared responsibility among stakeholders [55]; and institutional approaches (i.e., CRPs), which have been developed to address conflicts linked to end-of-life care, malpractice, and treatment futility through a formalized mediation process [1,10,35]. A smaller subset of the literature discussed training and education in mediation and the importance of equipping healthcare professionals with conflict resolution competencies. Such programs have been developed specifically for physicians and physician assistants [56,57]. Meanwhile, other studies reflected a growing institutional interest in embedding mediation competencies within clinical teams to prevent escalation and improve patient–provider relationships [58]. Last but not least, a growing body of work explored proactive or preventive ethics models as early warning tools, like team huddles and conflict scoring systems [30,59,60], that have the objective of identifying and defusing conflict before it escalates. The dominance of generic or conceptual mediation models may reflect the relative novelty of formalized mediation programs outside North America, where clinical ethics services have had decades to codify structured frameworks. In contrast, many European and Asian systems integrate mediation more flexibly into existing ethics committees, patient-rights structures, or administrative dispute-resolution pathways.

Within the legal and policy domain, most of the published literature addressed national statutes and legal frameworks [6,13], as well as hospital-wide mediation programs [31,37], while a substantial portion also explored the intersection of ethics and law, with emphasis on the role of mediation in the resolution of complex disputes on euthanasia [61], disclosure [18], and apology [32]. There is also strong evidence that CRPs reduce litigation and improve patient safety outcomes [1,5]. Finally, 16 publications discussed theoretical and normative aspects, challenging adversarial ethics-consultation norms [2,62], underpinning the role of common-morality in mediation [63] and the value of constructive disagreement [64], pluralism, and consensus building [65]. Notably, several European jurisdictions, including Italy, Spain, Portugal, France, and Greece, have integrated mediation within broader civil-law ADR reforms, which differ procedurally from Anglo-American tort-based approaches. These variations highlight that legal culture significantly shapes how mediation is institutionalized and how conflicts are framed at the health-system level.

Taken together, these findings indicate that the global landscape of medical mediation is characterized by both convergence and heterogeneity. While many systems draw on shared principles such as communication, impartial facilitation, and collaborative problem-solving, the structures through which mediation is enacted differ markedly across legal traditions, health-system designs, and cultural expectations about decision-making. This explains why some countries develop formalized mediation committees, while others rely on ethics consultation hybrids, and others integrate mediation within civil-law ADR statutes. Appreciating this variation is essential for interpreting the literature and prevents overgeneralization from any single national or conceptual model. It also underscores that effective medical mediation must be culturally and institutionally tailored. Rather than a weakness, this adaptability should be perceived as one of mediation’s core strengths, allowing the process to align with local norms, communication styles, and expectations of decision-making across different healthcare systems.

Medical mediation has been emerging as a core component of modern conflict resolution in healthcare due to not only its efficiency in resolving high-stakes disputes, but also its ability to reshape the culture of care through transparency, accountability, and relational ethics. The integration of structured mediation practices, supported by legal frameworks, clinical training, and evaluative research, seems to be a necessity for advancing both justice and compassion in clinical practice for healthcare systems that face increasing ethical complexity and institutional strain.

This review has the following limitations. First, the evidence base is naturally heterogeneous, reflecting the theoretical, narrative, and practice-based nature of the literature on medical mediation. Much of the literature consists of conceptual analyses, case reports, or descriptive accounts rather than rigorous empirical evaluations, which limits comparability and the ability to assess effectiveness across settings. Such diversity is characteristic of scientific fields that rely on experiential knowledge and normative argumentation. Hence, we employed JBI appraisal to ensure minimum methodological consistency. Second, despite independent screening and JBI assessment by two reviewers, the search was restricted to English-language publications and major databases, or studies with available English translations, which may have excluded relevant work from regions with strong mediation traditions or non-indexed program reports. These factors introduce potential publication bias and geographical imbalance and should be considered when interpreting the findings.

## 5. Conclusions

Medical mediation is an effective, patient-centered approach to resolving ethical and clinical disputes in healthcare. Over the past four decades, it has expanded from bedside applications to institutional and national frameworks, enhancing communication, trust, and fairness in clinical decision-making. Despite this progress, empirical evaluation and integration into training and policy remain limited. Strengthening legal frameworks and embedding mediation in ethics education can further promote justice, transparency, and compassion in healthcare systems.

## Data Availability

No new data were created or analyzed in this study. The data supporting the findings of this systematic review consist of previously published articles available in the PubMed and Scopus databases.

## Supplementary Materials

The following supporting information can be downloaded at: https://www.mdpi.com/article/10.3390/healthcare13243235/s1. Table S1: PRISMA 2020 Checklist for reporting systematic reviews. Table S2: JBI Critical Appraisal results for all 152 included studies [66,67,68,69,70,71,72,73,74,75,76,77,78,79,80,81,82,83,84,85,86,87,88,89,90,91,92,93,94,95,96,97,98,99,100,101,102,103,104,105,106,107,108,109,110,111,112,113,114,115,116,117,118,119,120,121,122,123,124,125,126,127,128,129,130,131,132,133,134,135,136,137,138,139,140,141,142,143,144,145,146,147,148,149,150,151,152,153,154,155,156,157].

## References

[1] Hyman C.S., Liebman C.B., Schechter C.B., Sage W.M. (2010). Interest-Based Mediation of Medical Malpractice Lawsuits: A Route to Improved Patient Safety?. J. Health Politics Policy Law.

[2] Fiester A. (2007). Mediation and Moral Aporia. J. Clin. Ethics.

[3] Dubler N.N. (2013). The Art of the Chart Note in Clinical Ethics Consultation and Bioethics Mediation: Conveying Information That Can Be Understood and Evaluated. J. Clin. Ethics.

[4] Dubler N.N., Liebman C.B. (2011). Bioethics Mediation: A Guide to Shaping Shared Solutions.

[5] Gallagher T.H., Mello M.M., Sage W.M., Bell S.K., McDonald T.B., Thomas E.J. (2018). Can Communication-And-Resolution Programs Achieve Their Potential? Five Key Questions. Health Aff..

[6] Wang M., Liu G.G., Zhao H., Butt T., Yang M., Cui Y. (2020). The Role of Mediation in Solving Medical Disputes in China. BMC Health Serv. Res..

[7] Page M.J., McKenzie J.E., Bossuyt P.M., Boutron I., Hoffmann T.C., Mulrow C.D., Shamseer L., Tetzlaff J.M., Akl E.A., Brennan S.E. (2021). The PRISMA 2020 Statement: An Updated Guideline for Reporting Systematic Reviews. BMJ.

[8] Munn Z., Barker T.H., Moola S., Tufanaru C., Stern C., McArthur A., Stephenson M., Aromataris E. (2020). Methodological Quality of Case Series Studies: An Introduction to the JBI Critical Appraisal Tool. JBI Database Syst. Rev. Implement. Rep..

[9] Craig K. (1993). Medical Mediation. Med. War.

[10] Sproule R. (1989). Update on the Medical Mediation Panels. Wis. Med. J..

[11] Kass J.S., Rose R.V. (2016). Medical Malpractice Reform--Historical Approaches, Alternative Models, and Communication and Resolution Programs. AMA J. Ethics.

[12] Metzloff T.B., Peeples R.A., Harris C.T. (1997). Empirical Perspectives on Mediation and Malpractice. Law Contemp. Probl..

[13] Kwon O.-T., Seon J.G., Kim S.Y. (2012). Discussions and Implications of the Recent Enactment & Revision of the Healthcare Law. J. Korean Med. Sci..

[14] Dubler N.N. (1998). Mediation and Managed Care. J. Am. Geriatr. Soc..

[15] Cheng K.K., le Roux-Kemp A. (2017). Mediation and Resolving Disputes Involving Emergency Nurses in Hong Kong: A Legal Empirical Inquiry. Hong Kong Law J..

[16] Selandari J.O., de la Portilla M., Ciruzzi M.S., Couceiro C., García H.O., Iervolino M.d.L.Á., Marín D.N., Miranda C., Novali L., Ortega L. (2022). Feasibility, effectiveness, and satisfaction achieved by the transdisciplinary intervention of a clinical-hospital ethics committee. A qualitative and quantitative study. Arch. Argent. Pediatr..

[17] Dimitrov K., Miteva-Katrandzhieva T. (2024). Mediation in Healthcare: Enhancing Conflict Resolution Between Patients and Physicians Beyond the Courtroom. Cureus.

[18] Chen P.-Y., Fu C.-P., Wang C.-C. (2023). Narratives in the Medicolegal Field from the Perspective of Physicians Involved in Medical Dispute Mediation Meetings in Taiwan. Heliyon.

[19] Nakanishi T. (2014). Disclosing Unavoidable Causes of Adverse Events Improves Patients’ Feelings towards Doctors. Tohoku J. Exp. Med..

[20] Mitello L. (2004). When the patient asks for counselling, when the patient doesn’t ask for counselling, when the patient refuses therapy. Prof. Inferm..

[21] Blanco Portillo A., García-Caballero R., Real de Asúa D., Olaciregui Dague K., Herreros B. (2024). What Ethics Support for Resolving Ethical Conflicts Do Internists Use in Spanish Hospitals?. J. Bioeth. Inq..

[22] Przylepa-Lewak A. (2023). Contemporary Challenges of Medical Mediation. Kryt. Prawa.

[23] Decoulx M., Scherpereel P. (2013). For a more humane hospital: Experience of medical mediators. Presse Med..

[24] Voultsos P., Tsompanian A., Tsaroucha A.K. (2021). The Medical Futility Experience of Nursing Professionals in Greece. BMC Nurs..

[25] Zanni A. (2014). Organ Transplantation in Greece: The Need for Mediation. Transplant. Proc..

[26] Díaz Rivera Y.A. (2015). Project for the Creation of a Medical or Hospital Ethical Committee at a Local Level in the San Miguel Arcangel Hospital, District of San Miguelito, Province of Panama. Year 2013. Cuad. Bioet..

[27] Ribeiro W.C. (2018). Mediation as a Means of Resolving Conflicts in the Healthcare Area. Rev. Direito Sanit..

[28] Dubler N.N. (2013). Commentary on Bergman: “Yes… But”. J. Clin. Ethics.

[29] DuBois J.M. (2024). The Bioethicist as Healer. Hastings Cent. Rep..

[30] Forbat L., Mnatzaganian G., Barclay S. (2019). The Healthcare Conflict Scale: Development, Validation and Reliability Testing of a Tool for Use across Clinical Settings. J. Interprof. Care.

[31] Hauschildt K., De Vries R. (2020). Reinforcing Medical Authority: Clinical Ethics Consultation and the Resolution of Conflicts in Treatment Decisions. Sociol. Health Illn..

[32] Gatter R. (2004). Institutionally Sponsored Mediation and the Emerging Medical Trust Movement in the U.S.. Med. Law.

[33] Regis C., Poitras J. (2010). Healthcare Mediation and the Need for Apologies. Health Law J..

[34] Nelson C.M., Nazareth B.A. (2013). Nonbeneficial Treatment and Conflict Resolution: Building Consensus. Perm. J..

[35] Powell T., Hulkower A. (2017). A Good Death. Hastings Cent. Rep..

[36] Singer P.A., Barker G., Bowman K.W., Harrison C., Kernerman P., Kopelow J., Lazar N., Weijer C., Workman S. (2001). Hospital Policy on Appropriate Use of Life-Sustaining Treatment. University of Toronto Joint Centre for Bioethics/Critical Care Medicine Program Task Force. Crit. Care Med..

[37] Meller S., Barclay S. (2011). Mediation: An Approach to Intractable Disputes between Parents and Paediatricians. Arch. Dis. Child..

[38] Miles F., Barclay S., Menson E., Shepherd T., Webster L. (2023). Boldly Going… Introducing Conflict Management Training to Starship Children’s Hospital. J. Paediatr. Child Health.

[39] Hoffman D.N., Strand G.R. (2024). “Sit down and Thrash It out”: Opportunities for Expanding Ethics Consultation during Conflict Resolution in Long-Term Care. New Bioeth..

[40] Savage T.A., Parson J., Zollman F., Kirschner K.L. (2009). Rehabilitation Team Disagreement: Guidelines for Resolution. PM R.

[41] Wood E., Karp N. (1994). Mediation: Reframing Care Conflicts in Nursing Homes. Generations.

[42] Geppert C.M.A., Shelton W. (2016). Health Care Ethics Committees as Mediators of Social Values and the Culture of Medicine. AMA J. Ethics.

[43] Kayser J.B., Kaplan L.J. (2020). Conflict Management in the ICU. Crit. Care Med..

[44] Chiarchiaro J., White D.B., Ernecoff N.C., Buddadhumaruk P., Schuster R.A., Arnold R.M. (2016). Conflict Management Strategies in the ICU Differ Between Palliative Care Specialists and Intensivists. Crit. Care Med..

[45] Forbat L., Teuten B., Barclay S. (2015). Conflict Escalation in Paediatric Services: Findings from a Qualitative Study. Arch. Dis. Child..

[46] Shen Y., Li G., Tang Z., Wang Q., Zhang Z., Hao X., Han X. (2024). Analysis of the Characteristics, Efficiency, and Influencing Factors of Third-Party Mediation Mechanisms for Resolving Medical Disputes in Public Hospitals in China. BMC Public Health.

[47] Gatter R. (1999). Unnecessary Adversaries at the End of Life: Mediating End-of-Life Treatment Disputes to Prevent Erosion of Physician-Patient Relationships. Boston Univ. Law Rev..

[48] Cline C. (2012). Why Some Conflicts Involving “‘difficult’ Patients” Should Remain Outside the Province of the Ethics Consultation Service. Am. J. Bioeth..

[49] Mondi A., Cozzi-Lepri A., Tavelli A., Cingolani A., Giacomelli A., Orofino G., De Girolamo G., Pinnetti C., Gori A., Saracino A. (2024). Persistent Poor Clinical Outcomes of People Living with HIV Presenting with AIDS and Late HIV Diagnosis—Results from the ICONA Cohort in Italy, 2009–2022. Int. J. Infect. Dis..

[50] Khushf G. (2019). When Religious Language Blocks Discussion About Health Care Decision Making. HEC Forum.

[51] Dubler N.N. (2002). Mediating Disputes in Managed Care: Resolving Conflicts over Covered Services. J. Health Care Law Policy.

[52] Madden S., Martin D.K., Downey S., Singer P.A. (2005). Hospital Priority Setting with an Appeals Process: A Qualitative Case Study and Evaluation. Health Policy.

[53] Fiester A. (2013). A Dubious Export: The Moral Perils of American-Style Ethics Consultation. Bioethics.

[54] Bergman E.J. (2015). Identifying Sources of Clinical Conflict: A Tool for Practice and Training in Bioethics Mediation. J. Clin. Ethics.

[55] Buchanan S.F., Desrochers J.M., Henry D.B., Thomassen G., Barrett P.H.J. (2002). A Mediation/Medical Advisory Panel Model for Resolving Disputes about End-of-Life Care. J. Clin. Ethics.

[56] Fournier V., Spranzi M., Foureur N., Brunet L. (2015). The “Commitment Model” for Clinical Ethics Consultations: Society’s Involvement in the Solution of Individual Cases. J. Clin. Ethics.

[57] Glover A., Bertino J. (2018). Bioethics Mediation: A Practical Approach to Physician Assistant Ethics Education. J. Physician Assist. Educ..

[58] Kayser J.B. (2015). Mediation Training for the Physician: Expanding the Communication Toolkit to Manage Conflict. J. Clin. Ethics.

[59] McGreevy J. (2015). In the Ethos of the Safety Net: An Expanded Role for Clinical Ethics Mediation. J. Clin. Ethics.

[60] Pavlish C., Brown-Saltzman K., Fine A., Jakel P. (2013). Making the Call: A Proactive Ethics Framework. HEC Forum.

[61] Yoo S.H., Kim Y., Choi W., Shin J., Kim M.S., Park H.Y., Keam B., Yim J.-J. (2023). Ethical Issues Referred to Clinical Ethics Support at a University Hospital in Korea: Three-Year Experience After Enforcement of Life-Sustaining Treatment Decisions Act. J. Korean Med. Sci..

[62] Martínez-López J.Á., Lozano E.B., Gómez P.M., Ayala J.A.G. (2023). The right to euthanasia: Mediation in end-of-life decision-making. Prism. Social..

[63] Dubler N.N. (2011). Commentary on Fiester’s “Ill-Placed Democracy: Ethics Consultations and the Moral Status of Voting. J. Clin. Ethics.

[64] Ahmadi Nasab Emran S. (2015). The Four-Principle Formulation of Common Morality Is at the Core of Bioethics Mediation Method. Med. Health Care Philos..

[65] Parker M.J. (2024). Bioethics and the Value of Disagreement. J. Med. Ethics.

[66] Martin P.A. (1999). Bioethics and the Whole: Pluralism, Consensus, and the Transmutation of Bioethical Methods into Gold. J. Law Med. Ethics.

[67] Ab Rahim S.F., Kusumaningrum A.E. (2025). Tort Litigation Versus Mediation in Medico-Legal Disputes: Evaluating The Limits Of Mediation and Proposals for Reform. J. Ftw. Mgt. Res..

[68] Alexander A.A. (2010). Complaints, Grievances, and Claims against Physicians: Does Tort Reform Make a Difference?. J. Healthc. Risk Manag..

[69] Alvarez Baranga M.J. (2023). Right and Duty of the Doctor and the Patient" Hospital Mediation: Reality or Utopia?. Louvain Med..

[70] Ando T. (2011). Healthcare Mediation Model for Nerologists. Rinsho Shinkeigaku = Clin. Neurol..

[71] Bergman E.J. (2013). A Response to Dubler’s Commentary on “Surmounting Elusive Barriers: The Case for Bioethics Mediation”. J. Clin. Ethics.

[72] Beyleveld D., Brownsword R., Wallace S. (2002). Clinical Ethics Committees: Clinician Support or Crisis Management?. HEC Forum.

[73] Bowen T. (2009). Using Mediation in Situations of Withholding or Withdrawing Life-Sustaining Treatment: A New South Wales Perspective. J. Law Med..

[74] Bowman K.W. (2000). Communication, Negotiation, and Mediation: Dealing with Conflict in End-of-Life Decisions. J. Palliat. Care.

[75] Brazg T., Lindhorst T., Dudzinski D., Wilfond B. (2016). Defining Patient Advocacy for the Context of Clinical Ethics Consultation: A Review of the Literature and Recommendations for Consultants. J. Clin. Ethics.

[76] Brown C.E., Marshall A.R., Cueva K.L., Snyder C.R., Kross E.K., Young B.A. (2024). Physician Perspectives on Addressing Anti-Black Racism. JAMA Netw. Open.

[77] Casarett D.J., Daskal F., Lantos J. (1998). The Authority of the Clinical Ethicist. Hastings Cent. Rep..

[78] Craig Y.J. (1996). Patient Decision-Making: Medical Ethics and Mediation. J. Med. Ethics.

[79] Crigger B.J. (1995). Negotiating the Moral Order: Paradoxes of Ethics Consultation. Kennedy Inst. Ethics J..

[80] DeAngelo L.M. (2000). Mediation in Health Care Settings: Some Theoretical and Practical Concepts. J. Clin. Psychol. Med. Settings.

[81] Dimitrov K.Y., Miteva-Katrandzhieva T. (2025). Exploring Patient Awareness and the Feasibility of Mediation in Healthcare: A Pilot Study in Bulgaria. Healthcare.

[82] Fiester A.M. (2011). Ill-Placed Democracy: Ethics Consultations and the Moral Status of Voting. J. Clin. Ethics.

[83] DuVal G., Sartorius L., Clarridge B., Gensler G., Danis M. (2001). What Triggers Requests for Ethics Consultations?. J. Med. Ethics.

[84] Eves M.M., Esplin B.S. (2015). “She Just Doesn’t Know Him Like We Do”: Illuminating Complexities in Surrogate Decision Making. J. Clin. Ethics.

[85] Felder R.M. (2024). Toward a New Clinical Pragmatism: Method in Clinical Ethics Consultation. Med. Health Care Philos..

[86] Fiester A. (2007). The Failure of the Consult Model: Why “Mediation” Should Replace “Consultation”. Am. J. Bioeth..

[87] Fiester A. (2012). Mediation and Advocacy. Am. J. Bioeth..

[88] Fiester A. (2015). Neglected Ends: Clinical Ethics Consultation and the Prospects for Closure. Am. J. Bioeth..

[89] Fiester A. (2024). The “Ladder of Inference” as a Conflict Management Tool: Working with the “Difficult” Patient or Family in Healthcare Ethics Consultations. HEC Forum.

[90] Fiester A. (2025). Defending Dubler’s Legacy: Relocating the Role of Conflict Management from the Ethics Consultation Service to Patient and Guest Relations. J. Clin. Ethics.

[91] Fiester A. (2025). The Transformative Power of Reasons Relitigates Concerns about Non-Facilitated Healthcare Ethics Consultation. Am. J. Bioeth..

[92] Flicker L., Powell T. (2025). The House That Nancy Built. J. Clin. Ethics.

[93] Forbat L., Barclay S. (2019). Reducing Healthcare Conflict: Outcomes from Using the Conflict Management Framework. Arch. Dis. Child..

[94] Forbat L., Simons J., Sayer C., Davies M., Barclay S. (2017). Training Paediatric Healthcare Staff in Recognising, Understanding and Managing Conflict with Patients and Families: Findings from a Survey on Immediate and 6-Month Impact. Arch. Dis. Child..

[95] François K., Lobb E., Barclay S., Forbat L. (2017). The Nature of Conflict in Palliative Care: A Qualitative Exploration of the Experiences of Staff and Family Members. Patient Educ. Couns..

[96] Gibson J.M. (1994). Mediation for Ethics Committees: A Promising Process. Generations.

[97] Gibson K. (1999). Mediation in the Medical Field. Is Neutral Intervention Possible?. Hastings Cent. Rep..

[98] Goranova-Spasova R., Gradinarova N. (2022). Application of mediation in the field of healthcare. General Med..

[99] Greco E. (2025). Disputing Death: Medical Futility Laws and Procedures to Facilitate End of Life Discussions among Patients, Family, and Practitioners. SDL Rev..

[100] Herron P.D. (2025). Honoring Chosen Family: Revisiting the Doctor-Proxy Relationship. J. Clin. Ethics.

[101] Howe E.G. (2025). Nancy Dubler’s Contributions to Clinical Ethics Consultation. J. Clin. Ethics.

[102] Johal H.K., Birchley G., Huxtable R. (2022). Exploring Physician Approaches to Conflict Resolution in End-of-Life Decisions in the Adult Intensive Care Unit: Protocol for a Systematic Review of Qualitative Research. BMJ Open.

[103] Kolak J., Hulkower A. (2025). Dialogic Engagement and the Epistemic Norms of Bioethics Mediation. J. Clin. Ethics.

[104] Latham S. (2015). Facilitated Discussion: Good and Good for You. Am. J. Bioeth..

[105] Lee D.W.H., Lai P.B.S. (2015). The Practice of Mediation to Resolve Clinical, Bioethical, and Medical Malpractice Disputes. Hong Kong Med. J..

[106] Lindsey J., Doyle M., Wazynska-Finck K. (2025). Securing Therapeutic Justice through Mediation: The Challenge of Medical Treatment Disputes. Leg. Stud..

[107] Lindsey J., Francis G., Doyle M. (2025). Mediation of Medical Treatment Disputes: A Therapeutic Justice Model End of Project Report.

[108] Linney M., Hain R.D.W., Wilkinson D., Fortune P.-M., Barclay S., Larcher V., Fitzgerald J., Arkell E. (2019). Achieving Consensus Advice for Paediatricians and Other Health Professionals: On Prevention, Recognition and Management of Conflict in Paediatric Practice. Arch. Dis. Child..

[109] Lyons O., Forbat L., Menson E., Chisholm J.C., Pryde K., Conlin S., Felton V., Ingle S., McKenzie C., Ramachandran R. (2021). Transforming Training into Practice with the Conflict Management Framework: A Mixed Methods Study. BMJ Paediatr. Open.

[110] Matchett N.J. (2015). Philosophical Counseling as an Alternative Process to Bioethics Mediation. Am. J. Bioeth..

[111] Maung A.A., Toevs C.C., Kayser J.B., Kaplan L.J. (2015). Conflict Management Teams in the Intensive Care Unit: A Concise Definitive Review. J. Trauma Acute Care Surg..

[112] McClimans L., Pressgrove G., Campbell E. (2019). Objectives and Outcomes of Clinical Ethics Services: A Delphi Study. J. Med. Ethics.

[113] Miller R.B. (2002). Extramural Ethics Consultation: Reflections [Correction of Relections] on the Mediation/Medical Advisory Panel Model and a Further Proposal. J. Clin. Ethics.

[114] Morreim H. (2015). Conflict Resolution in the Clinical Setting: A Story Beyond Bioethics Mediation. J. Law Med. Ethics.

[115] Morreim H. (2024). From Philosopher in Residence to Healthcare Mediation. J. Law Med. Ethics.

[116] Morreim E.H. (2025). A Great Gift … with Great Caveats: Bioethics Mediation versus Bona Fide Mediation. J. Clin. Ethics.

[117] Munuera Gómez P. (2020). Medical mediation in the international context. Rev. Med. Chil..

[118] Nicolas P., Sullivan L.S., Chuang E. (2025). Mediation of Medical Distrust Due to Racial Injustice: The Legacy of Nancy Dubler. J. Clin. Ethics.

[119] Oh E.H., Shin J.E., Bae J.Y., Lee Y.S., Park Y., Kwon Y.H., Paik C.N., Lee J.K., Lee T.H. (2025). Medical Disputes Involving Lower Gastrointestinal Endoscopies: Cases from the Korean Medical Dispute Mediation and Arbitration Agency. Korean J. Intern. Med..

[120] Omelianchuk A., Ansari A.A., Parsi K. (2024). What Is It That You Want Me To Do? Guidance for Ethics Consultants in Complex Discharge Cases. HEC Forum.

[121] Ong C. (2013). Medical Mediation: Bringing Everyone to the Table. Bull. Am. Coll. Surg..

[122] Orr R.D., deLeon D.M. (2000). The Role of the Clinical Ethicist in Conflict Resolution. J. Clin. Ethics.

[123] Orr R.D., Marshall P.A., Osborn J. (1995). Cross-Cultural Considerations in Clinical Ethics Consultations. Arch. Fam. Med..

[124] Orr R.D. (2001). Methods of Conflict Resolution at the Bedside. Am. J. Bioeth..

[125] Orr R.D. (2002). Working toward Peace in the Clinical Setting: The Role of Clinical Ethics in Conflict Resolution. Todays Christ. Dr..

[126] Paquette E.T., Kolaitis I.N. (2015). Intensive Care, Intense Conflict: A Balanced Approach. J. Clin. Ethics.

[127] Perangin-Angin T.A., Silaban L.S., Batubara S.A., Sinaga J. (2025). Mediation as an Alternative to Legal Dispute Resolution in Health Services in Hospitals. JUSTISI.

[128] Phillipson J., Barclay S., Menson E., Lyons O. (2024). Healthcare Decision-Makers’ Perspectives on Evaluating Conflict Management Training in Paediatric Healthcare: A Utilisation-Focused Qualitative Study. BMJ Paediatr. Open.

[129] Phua J. (2022). Healthcare Mediation: Bridging the Gap. Contemporary Issues in Mediation: Volume 7.

[130] Rangkutir R., Risdawati I. (2024). The role of hospital management in resolution of medical disputes through mediation paths in the hospital. Int. Conf. Health Sci. Green Econ. Educ. Rev. Technol..

[131] Reynolds D.F. (1994). Consultectonics: Ethics Committee Case Consultation as Mediation. Bioeth. Forum.

[132] Rotily M., Lamouroux-Delay A., Cristina Rojas-Vergara A. (2025). Recours à La Médiation En Santé Chez Les Patients En Situation de Précarité: L’exemple Du Recours Aux Urgences. Santé Publique.

[133] Saito Y., Takeda K., Akama N., Yamauchi T. (2012). Significance of Training in Healthcare Mediation to Resolve Conflicts between Health Professionals and Patients. Jpn. J. Natl. Med. Serv..

[134] Sari I. (2025). Alternative Dispute Resolution In Medical Dispute Resolution: Initiating The Establishment Of An Alternative Medical Dispute Resolution Institution In Indonesia. Fox Justi J. Ilmu Huk..

[135] Schildmann J., Nadolny S., Haltaufderheide J., Gysels M., Vollmann J., Bausewein C. (2019). Do We Understand the Intervention? What Complex Intervention Research Can Teach Us for the Evaluation of Clinical Ethics Support Services (CESS). BMC Med. Ethics.

[136] Schlairet M.C. (2009). Bioethics Mediation: The Role and Importance of Nursing Advocacy. Nurs. Outlook.

[137] Scofield G.R. (1995). In Medias Res: The Ethicist as Mediator. Trends Health Care Law Ethics.

[138] Sriwidodo J., Wahid S.H., Kususiyanah A. (2025). Toward Equitable Healthcare: A Medical Dispute Resolution Framework to Address Medical Supply Delays in Health Law. J. Leg. Aff. Disput. Resolut. Eng. Constr..

[139] Stevenson J., Clinch A., Ftanou M., Delany C. (2024). What Is Known about the Role of Clinical Ethics Services in Cancer Care? A Systematic/Narrative Literature Review. BMJ Support. Palliat. Care.

[140] Tan H.S. (2025). What’s Best and Who Decides for Seriously Ill Infants? A Malaysian Perspective. Asian Bioeth. Rev..

[141] Tantiono P., Darma I.M.W., Kurniawan I.G.A. (2025). The Rights of Families of Terminal Patients to Refuse Futile Treatment: Legal and Ethical Limitations. J. Pena Justisia.

[142] Teremetskyi V., Tokarieva K., Batryn O., Myrza S., Mosondz S., Matviichuk A. (2024). Mediation as an Effective Mechanism for Resolving Disputes Caused by Medical Errors. Azerbaijan Pharm. Pharmacother. J..

[143] Trotter G. (2002). Bioethics and Healthcare Reform: A Whig Response to Weak Consensus. Camb. Q. Healthc. Ethics.

[144] Turner K. (2024). Hidden Fault Lines in the Bedrock: A Critical Examination of Surrogate Decision-Making Standards in Ethics Consultation. J. Clin. Ethics.

[145] Wada Y. (2012). Suggestions from Sites of Healthcare Mediation. IRYO-Jpn. J. Natl. Med. Serv..

[146] Waldman E. (2003). Mediating Difference: Normative Conflict as Opportunity. Am. J. Bioeth..

[147] Walton M.K. (2015). Patient-Centered Care and the Mediator’s Skills. J. Clin. Ethics.

[148] Watkins L.T., Sacajiu G., Karasz A. (2007). The Role of the Bioethicist in Family Meetings about End of Life Care. Soc. Sci. Med..

[149] Weaver M.S., Boss R.D., Christopher M.J., Gray T.F., Harman S., Madrigal V.N., Michelson K.N., Paquette E.T., Pentz R.D., Scarlet S. (2022). Top Ten Tips Palliative Care Clinicians Should Know About Their Work’s Intersection with Clinical Ethics. J. Palliat. Med..

[150] Weinstein M.S. (2015). A Second Opinion: A Case Narrative on Clinical Ethics Mediation. J. Clin. Ethics.

[151] Welie J.V. (1998). Clinical Ethics: Theory or Practice?. Theor. Med. Bioeth..

[152] West M.B., Gibson J.M. (1992). Facilitating Medical Ethics Case Review: What Ethics Committees Can Learn from Mediation and Facilitation Techniques. Camb. Q. Healthc. Ethics.

[153] Widjaja G. (2025). Settlement of medical disputes due to minor offences by health workers through mediation. INJOSEDU Int. J. Soc. Educ..

[154] Wilkinson D., Barclay S., Savulescu J. (2018). Disagreement, Mediation, Arbitration: Resolving Disputes about Medical Treatment. Lancet.

[155] Wright L., Ross K., Daar A.S. (2005). The Roles of a Bioethicist on an Organ Transplantation Service. Am. J. Transplant..

[156] Zákány J. (2025). Alternative dispute resolution in healthcare sector in Hungary: The role of conciliation boards. Curentul Jurid..

[157] Zhang J. (2025). Reevaluating Benevolent Deception: A Trust-Oriented Approach to Ethical Mediation in Multicultural Healthcare. J. Clin. Ethics.

