# Supplementary material for: Applications of Medical Mediation: A Systematic Review of Its Role in Healthcare Dispute Resolution and Bioethical Decision-Making
Source: Healthcare (Basel). 2025 Dec 10;13(24):3235. doi: 10.3390/healthcare13243235 (PMC12732525; doi:10.3390/healthcare13243235)
Supplement: Supplementary file 1 [file healthcare-13-03235-s001.zip › TableS2_R1.pdf]

**Title**	**Reference**	**JBI Inclusion justification**
Tort Litigation Versus Mediation in Medico-Legal Disputes: Evaluating The Limits Of Mediation and Proposals for Reform	(Ab Rahim and Kusumaningrum, 2025)	Include (Text & Opinion)
The four-principle formulation of common morality is at the core of bioethics mediation method	(Ahmadi Nasab Emran, 2015)	Include (Text & Opinion evidence)
Complaints, grievances, and claims against physicians: does tort reform make a difference?	(Alexander, 2010)	Include (Descriptive/Contextual quantitative evidence)
Right and duty of the doctor and the patient: Hospital mediation: reality or utopia?	(Alvarez Baranga, 2023)	Include (Case report)
Healthcare mediation model for neurologists	(Ando, 2011)	Include (Text & Opinion evidence)
A response to Dubler's commentary on "surmounting elusive barriers: the case for bioethics mediation"	(Bergman, 2013)	Include (Text & Opinion evidence)
Identifying Sources of Clinical Conflict: A Tool for Practice and Training in Bioethics Mediation	(Bergman, 2015)	Include (Text & Opinion evidence)
Clinical ethics committees: clinician support or crisis management?	(Beyleveld et al., 2002)	Include (Text & Opinion evidence)
What Ethics Support for Resolving Ethical Conflicts Do Internists Use in Spanish Hospitals?	(Blanco Portillo et al., 2024)	Include (Analytical Cross-Sectional)

Using mediation in situations of withholding or withdrawing life-sustaining treatment: a New South Wales perspective	(Bowen, 2009)	Include (Text & Opinion evidence)
Communication, negotiation, and mediation: dealing with conflict in end-of-life decisions	(Bowman, 2000)	Include (Text & Opinion evidence)
Defining Patient Advocacy for the Context of Clinical Ethics Consultation	(Brazg et al., 2016)	Include (Text & Opinion evidence)
Physician Perspectives on Addressing Anti-Black Racism	(Brown et al., 2024)	Include (Qualitative evidence)
A mediation/medical advisory panel model for resolving disputes about end-of-life care	(Buchanan et al., 2002)	Include (Qualitative / descriptive evidence relevant to mediation models)
The authority of the clinical ethicist	(Casarett et al., 1998)	Include (Text & Opinion evidence)
Narratives in the medicolegal field ... medical dispute mediation meetings in Taiwan	(Chen et al., 2023)	Include (Qualitative evidence)
Mediation and resolving disputes involving emergency nurses in Hong Kong: A legal empirical inquiry	(Cheng and le Roux-Kemp, 2017)	Include (Qualitative / descriptive evidence relevant to mediation models)
Conflict Management Strategies in the ICU Differ Between Palliative Care Specialists and Intensivists	(Chiarchiaro et al., 2016)	Include (High-quality qualitative observational evidence)
Why some conflicts involving "'difficult' patients" should remain outside the	(Cline, 2012)	Include (Conceptual qualitative evidence relevant to ethics mediation boundaries)

province of the ethics consultation service		
Medical mediation	(Craig, 1993)	Include with caution (Low methodological rigour, high contextual relevance).
Patient decision-making: medical ethics and mediation	(Craig, 1996)	Include (Text & Opinion)
Negotiating the moral order: paradoxes of ethics consultation	(Crigger, 1995)	Include (Text & Opinion)
Mediation in health care settings: Some theoretical and practical concepts	(DeAngelo, 2000)	Include (Text & Opinion Evidence)
[For a more humane hospital: experience of medical mediators]	(Decoulx and Scherpereel, 2013)	Include (Text & Opinion Evidence)
[Project for the Creation of a Medical or Hospital Ethical Committee ... in Panama]	(Díaz Rivera, 2015)	Include (Cross sectional study)
Mediation in Healthcare: Enhancing Conflict Resolution Between Patients and Physicians Beyond the Courtroom	(Dimitrov and Miteva-Katrandzhieva, 2024)	Include (Conceptual/Text evidence)
Exploring Patient Awareness and the Feasibility of Mediation in Healthcare: A Pilot Study in Bulgaria	(Dimitrov and Miteva-Katrandzhieva, 2025)	Include (Cross-sectional Pilot Study)
Mediation and managed care	(Dubler, 1998)	Include (Text & Opinion evidence)
Mediating disputes in managed care: resolving conflicts over covered services	(Dubler, 2002)	Include (Text & Opinion evidence)
Ill-placed democracy: ethics consultations and the moral status of voting	(Fiester, 2011)	Include (Conceptual/Theoretical Evidence)

Commentary on Fiester's "Ill-placed democracy: ethics consultations and the moral status of voting"	(Dubler, 2011)	Include (high-value conceptual evidence on mediation in ethics consultation)
The art of the chart note in clinical ethics consultation and bioethics mediation	(Dubler, 2013a)	Include (valuable conceptual/practice evidence)
Commentary on Bergman: "yes… but"	(Dubler, 2013b)	Include (Conceptual / theoretical evidence)
The Bioethicist as Healer	(DuBois, 2024)	Include (Conceptual/Theoretical Evidence)
What triggers requests for ethics consultations?	(DuVal et al., 2001)	Include (Cross sectional study)
"She Just Doesn't Know Him Like We Do": Illuminating Complexities in Surrogate Decision Making	(Eves and Esplin, 2015)	Include (Text & opinion evidence)
Toward a new clinical pragmatism: method in clinical ethics consultation	(Felder, 2024)	Include (Text & opinion evidence)
Mediation and moral aporia	(Fiester, 2007a)	Include (Text & opinion evidence)
The failure of the consult model: why "mediation" should replace "consultation"	(Fiester, 2007b)	Include (Conceptual / Normative evidence)
Mediation and advocacy	(Fiester, 2012)	Include (Conceptual/Theoretical Evidence)
A dubious export: the moral perils of American-style ethics consultation	(Fiester, 2013)	Include (High-quality Text & Opinion evidence)
Neglected ends: clinical ethics consultation and the prospects for closure	(Fiester, 2015)	Include (High-quality Text & Opinion evidence)

The “Ladder of Inference” as a Conflict Management Tool … Healthcare Ethics Consultations	(Fiester, 2024)	Include (High-quality Text & Opinion evidence)
Defending Dubler’s Legacy: Relocating the Role of Conflict Management from the Ethics Consultation Service to Patient and Guest Relations	(Fiester, 2025a)	Include (Text & Opinion)
The Transformative Power of Reasons Relitigates Concerns about Non-Facilitated Healthcare Ethics Consultation	(Fiester, 2025b)	Include (Text & Opinion)
The House That Nancy Built	(Flicker and Powell, 2025)	Include (Text & Opinion)
Reducing healthcare conflict: outcomes from using the conflict management framework	(Forbat and Barclay, 2019)	Include (Quasi-Experimental evidence)
Conflict escalation in paediatric services: findings from a qualitative study	(Forbat et al., 2015)	Include (High-quality qualitative evidence)
Training paediatric healthcare staff in recognising, understanding and managing conflict with patients and families	(Forbat et al., 2017)	Include (Quasi-Experimental evidence)
The “Commitment Model” for Clinical Ethics Consultations	(Fournier et al., 2015)	Include (High-value expert opinion / conceptual evidence)
The nature of conflict in palliative care: A qualitative exploration of the experiences of staff and family members	(François et al., 2017)	Include (High-quality qualitative evidence)

Can Communication-And-Resolution Programs Achieve Their Potential? Five Key Questions	(Gallagher et al., 2018)	Include (High relevance as expert evidence)
Unnecessary adversaries at the end of life: mediating end-of-life treatment disputes to prevent erosion of physician-patient relationships	(Gatter, 1999)	Include (Text & Opinion evidence)
Institutionally sponsored mediation and the emerging medical trust movement in the U.S.	(Gatter, 2004)	Include (Text & Opinion evidence)
Health Care Ethics Committees as Mediators of Social Values and the Culture of Medicine	(Geppert and Shelton, 2016)	Include (Text & Opinion evidence)
Mediation for ethics committees: a promising process	(Gibson, 1994)	Include (Text & Opinion evidence)
Mediation in the medical field. Is neutral intervention possible?	(Gibson, 1999)	Include (Text & Opinion evidence)
Bioethics Mediation: A Practical Approach to Physician Assistant Ethics Education	(Glover and Bertino, 2018)	Include (High-quality Text & Opinion evidence)
APPLICATION OF MEDIATION IN THE FIELD OF HEALTHCARE	(Goranova-Spasova and Gradinarova, 2022)	Include (Analytical Cross-Sectional)
Disputing Death: Medical Futility Laws and Procedures to Facilitate End of Life Discussions among Patients, Family, and Practitioners	(Greco, 2025)	Include (Text & Opinion)
Reinforcing medical authority: clinical ethics consultation and the resolution of conflicts in treatment decisions	(Hauschildt and De Vries, 2020)	Include (Qualitative evidence)

Honoring Chosen Family: Revisiting the Doctor-Proxy Relationship	(Herron, 2025)	Include (Text & Opinion)
'Sit down and thrash it out': opportunities for expanding ethics consultation	(Hoffman and Strand, 2024)	Include (Analytical Cross-Sectional)
Nancy Dubler's Contributions to Clinical Ethics Consultation	(Howe, 2025)	Include (Text & Opinion)
Interest-based mediation of medical malpractice lawsuits: a route to improved patient safety?	(Hyman et al., 2010)	Include (Qualitative evidence)
Exploring physician approaches to conflict resolution in end-of-life decisions in the adult intensive care unit	(Johal et al., 2022)	Include (Qualitative evidence)
Medical Malpractice Reform–Historical Approaches, Alternative Models, and Communication and Resolution Programs	(Kass and Rose, 2016)	*Include as conceptual/theoretical evidence*
Conflict Management in the ICU	(Kayser and Kaplan, 2020)	Include (Text & Opinion evidence)
Mediation Training for the Physician: Expanding the Communication Toolkit to Manage Conflict	(Kayser, 2015)	*Include as conceptual/theoretical evidence*
When Religious Language Blocks Discussion About Health Care Decision Making	(Khushf, 2019)	Include (Text & Opinion evidence)
Dialogic Engagement and the Epistemic Norms of Bioethics Mediation	(Kolak and Hulkower, 2025)	Include (Conceptual / theoretical evidence)

Discussions and implications of the recent enactment & revision of the healthcare law	(Kwon et al., 2012)	Include (Text & Opinion evidence)
Facilitated discussion: good and good for you	(Latham, 2015)	Include
The practice of mediation to resolve clinical, bioethical, and medical malpractice disputes	(Lee and Lai, 2015)	Include (Text & Opinion evidence)
Securing therapeutic justice through mediation: the challenge of medical treatment disputes	(Lindsey et al., 2025a)	Include (Text & Opinion)
Mediation of medical treatment disputes: a therapeutic justice model end of project report	(Lindsey et al., 2025b)	Include (Qualitative evidence)
Achieving consensus advice for paediatricians …	(Linney et al., 2019)	Include (Text & Opinion evidence)
Transforming training into practice with the conflict management framework: a mixed methods study	(Lyons et al., 2021)	Include (Text & Empirical evidence)
Hospital priority setting with an appeals process: a qualitative case study and evaluation	(Madden et al., 2005)	Include (Text & Empirical evidence)
Bioethics and the whole: pluralism, consensus, and the transmutation of bioethical methods into gold	(Martin, 1999)	Include (Text & Empirical evidence)
The Right to Euthanasia: Mediation in End-of-Life Decision-Making	(Martínez-López et al., 2023)	Include (Empirical quantitative study – health-care mediation evidence)

Philosophical counseling as an alternative process to bioethics mediation	(Matchett, 2015)	Include (Text & Opinion evidence)
Conflict management teams in the intensive care unit: A concise definitive review	(Maung et al., 2015)	Include (Text & Opinion evidence)
Objectives and outcomes of clinical ethics services: a Delphi study	(McClimans et al., 2019)	Include (Text & Opinion evidence)
In the Ethos of the Safety Net: An Expanded Role for Clinical Ethics Mediation	(McGreevy, 2015)	Include (Text & Opinion evidence)
Mediation: an approach to intractable disputes between parents and paediatricians	(Meller and Barclay, 2011)	Include (Text & Opinion evidence)
Boldly going… Introducing conflict management training to Starship Children's Hospital	(Miles et al., 2023)	Include (Text & Opinion evidence)
Extramural ethics consultation: reflections on the mediation/medical advisory panel model and a further proposal	(Miller, 2002)	Include (Text & Opinion evidence)
[When the patient asks for counseling, when the patient doesn't ask for counseling, when the patient refuses therapy]	(Mitello, 2004)	Include (Text & Opinion evidence)
Conflict Resolution in the Clinical Setting: A Story Beyond Bioethics Mediation	(Morreim, 2015)	Include (Text & Opinion evidence)

From Philosopher in Residence to Healthcare Mediation	(Morreim, 2024)	Include (Text & Opinion evidence)
A Great Gift ... with Great Caveats: Bioethics Mediation versus Bona Fide Mediation	(Morreim, 2025)	Include (Text & Opinion)
[Medical mediation in the international context]	(Munuera Gómez, 2020)	Include (Text & Opinion evidence)
Disclosing unavoidable causes of adverse events improves patients' feelings towards doctors	(Nakanishi, 2014)	Include (Empirical evidence)
Nonbeneficial treatment and conflict resolution: building consensus	(Nelson and Nazareth, 2013)	Include (Empirical evidence)
Mediation of Medical Distrust due to Racial Injustice: The Legacy of Nancy Dubler	(Nicolas et al., 2025)	Include (Text & Opinion)
Medical disputes involving lower gastrointestinal endoscopies: cases from the Korean Medical Dispute Mediation and Arbitration Agency	(Oh et al., 2025)	Include (Analytical Case Series)
What Is It That You Want Me To Do? Guidance for Ethics Consultants in Complex Discharge Cases	(Omelianchuk et al., 2024)	Include (Text & Opinion evidence)
Medical mediation: bringing everyone to the table	(Ong, 2013)	Include (Text & Opinion evidence)
The role of the clinical ethicist in conflict resolution	(Orr and deLeon, 2000)	Include (Text & Opinion evidence)
Cross-cultural considerations in clinical ethics consultations	(Orr et al., 1995)	Include (Text & Opinion evidence)

Methods of conflict resolution at the bedside	(Orr, 2001)	Include (Text & Opinion evidence)
Working toward peace in the clinical setting: the role of clinical ethics in conflict resolution	(Orr, 2002)	Include (Text & Opinion evidence)
Intensive Care, Intense Conflict: A Balanced Approach	(Paquette and Kolaitis, 2015)	Include (Text & Opinion evidence)
Bioethics and the value of disagreement	(Parker, 2024)	Include (Text & Opinion evidence)
Making the call: a proactive ethics framework	(Pavlish et al., 2013)	Include (Text & Opinion evidence)
Mediation as an Alternative to Legal Dispute Resolution in Health Services in Hospitals	(Perangin-Angin et al., 2025)	Include (Text & Opinion)
Healthcare decision-makers' perspectives on evaluating conflict management training in paediatric healthcare	(Phillipson et al., 2024)	Include (Text & Opinion evidence)
Healthcare Mediation: Bridging the Gap	(Phua, 2022)	Include (Text & Opinion evidence)
A Good Death	(Powell and Hulkower, 2017)	Include (Text & Opinion evidence)
Contemporary challenges of medical mediation	(Przylepa-Lewak, 2023)	Include (Text & Opinion evidence)
THE ROLE OF HOSPITAL MANAGEMENT IN RESOLUTION OF MEDICAL DISPUTES THROUGH MEDIATION PATHS IN THE HOSPITAL	(Rangkutir and Risdawati, 2024)	Include (Text & Opinion)

Healthcare mediation and the need for apologies	(Regis and Poitras, 2010)	Include (Text & Opinion evidence)
Consultectonics: ethics committee case consultation as mediation	(Reynolds, 1994)	Include (Text & Opinion evidence)
Mediation as a means of resolving conflicts in the healthcare area	(Ribeiro, 2018)	Include (Text & Opinion evidence)
Recours à la médiation en santé chez les patients en situation de précarité : l'exemple du recours aux urgences	(Rotily et al., 2025)	Include (Text & Opinion / Narrative Review)
Significance of training in healthcare mediation to resolve conflicts between health professionals and patients	(Saito et al., 2012)	Include (Text & Opinion evidence)
Alternative Dispute Resolution In Medical Dispute Resolution: Initiating The Establishment Of An Alternative Medical Dispute Resolution Institution In Indonesia	(Sari, 2025)	Include (Text & Opinion)
Rehabilitation team disagreement: guidelines for resolution	(Savage et al., 2009)	Include (Text & Opinion evidence)
Do we understand the intervention? What complex intervention research can teach us for the evaluation of clinical ethics support services (CESS)	(Schildmann et al., 2019)	Include (Text & Opinion evidence)
Bioethics mediation: the role and importance of nursing advocacy	(Schlairet, 2009)	Include (Text & Opinion evidence)
In medias res: the ethicist as mediator	(Scofield, 1995)	Include (Text & Opinion evidence)

Feasibility, effectiveness, and satisfaction achieved by the transdisciplinary intervention of a clinical-hospital ethics committee	(Selandari et al., 2022)	Include (Empirical evidence – Mixed methods)
Analysis of the characteristics, efficiency, and influencing factors of third-party mediation...	(Shen et al., 2024)	Include (Empirical evidence - Quantitative)
Hospital policy on appropriate use of life-sustaining treatment	(Singer et al., 2001)	Include (Text & Opinion evidence)
Update on the medical mediation panels	(Sproule, 1989)	Include (Text & Opinion evidence)
Toward Equitable Healthcare: A Medical Dispute Resolution Framework to Address Medical Supply Delays in Health Law	(Sriwidodo et al., 2025)	Include (Text & Opinion)
What is known about the role of clinical ethics services in cancer care?	(Stevenson et al., 2024)	Include (Text & Opinion + Narrative Review evidence)
What's Best and Who Decides for Seriously Ill Infants? A Malaysian Perspective	(Tan, 2025)	Include (Text & Opinion)
The Rights of Families of Terminal Patients to Refuse Futile Treatment	(Tantiono et al., 2025)	Include (Text & Opinion)
Mediation as an Effective Mechanism for Resolving Disputes Caused by Medical Errors	(Teremetskyi et al., 2024)	Include (Text & Opinion)
The Healthcare Conflict Scale: development, validation and reliability testing of a tool for use across clinical settings	(*The Healthcare Conflict Scale: development, validation and reliability testing of a tool for use across clinical settings.*, 2019)	Include (High-quality psychometric evidence)

Bioethics and healthcare reform: a Whig response to weak consensus	(Trotter, 2002)	Include (Text & Opinion evidence)
Hidden Fault Lines in the Bedrock: A Critical Examination of Surrogate Decision-Making Standards	(Turner, 2024)	Include (Text & Opinion evidence)
The medical futility experience of nursing professionals in Greece	(Voultsos et al., 2021)	Include (Qualitative Text evidence)
Suggestions from sites of healthcare mediation	(Wada, 2012)	Include (Text & Opinion evidence)
Mediating difference: normative conflict as opportunity	(Waldman, 2003)	Include (Text evidence, conceptual analysis)
Patient-Centered Care and the Mediator's Skills	(Walton, 2015)	Include (Text evidence, conceptual & practice-based evidence)
The role of mediation in solving medical disputes in China	(Wang et al., 2020)	Include (Text evidence, empirical health-system evidence)
The role of the bioethicist in family meetings about end-of-life care	(Watkins et al., 2007)	Include (Text evidence – observational, mediation-focused evidence)
Top Ten Tips Palliative Care Clinicians Should Know About Their Work's Intersection with Clinical Ethics	(Weaver et al., 2022)	Include (Text evidence – conceptual & practice-oriented)
A Second Opinion: A Case Narrative on Clinical Ethics Mediation	(Weinstein, 2015)	Include (Text evidence – mediation-focused case narrative)
Clinical ethics: theory or practice?	(Welie, 1998)	Include (Conceptual/theoretical evidence)
Facilitating medical ethics case review: what ethics committees can learn from mediation and facilitation techniques	(West and Gibson, 1992)	Include (Text & Opinion evidence)

SETTLEMENT OF MEDICAL DISPUTES DUE TO MINOR OFFENCES BY HEALTH WORKERS THROUGH MEDIATION	(Widjaja, 2025)	Include (Text & Opinion)
Disagreement, mediation, arbitration: resolving disputes about medical treatment	(Wilkinson et al., 2018)	Include (Text & Opinion evidence)
Mediation: reframing care conflicts in nursing homes	(Wood and Karp, 1994)	Include (Text & Opinion evidence)
The roles of a bioethicist on an organ transplantation service	(Wright et al., 2005)	Include (Text & Opinion evidence)
Ethical Issues Referred to Clinical Ethics Support at a University Hospital in Korea	(Yoo et al., 2023)	Include (Text & Opinion evidence)
ALTERNATIVE DISPUTE RESOLUTION IN HEALTHCARE SECTOR IN HUNGARY: THE ROLE OF CONCILIATION BOARDS	(Zákány, 2025)	Include (Text & Opinion)
Organ transplantation in Greece: the need for mediation	(Zanni, 2014)	Include (Text & Opinion evidence)
Reevaluating Benevolent Deception: A Trust-Oriented Approach to Ethical Mediation in Multicultural Healthcare	(Zhang, 2025)	Include (Text & Opinion)

References

1. Ab Rahim, S.F., Kusumaningrum, A.E., 2025. Tort Litigation Versus Mediation in Medico-Legal Disputes: Evaluating The Limits Of Mediation and Proposals for Reform. j. ftw. mgt. res. 30, 176–197. https://doi.org/10.33102/jfatwa.vol30no2.710
2. Ahmadi Nasab Emran, S., 2015. The four-principle formulation of common morality is at the core of bioethics mediation method. Med Health Care Philos 18, 371–377. https://doi.org/10.1007/s11019-014-9612-7
3. Alexander, A.A., 2010. Complaints, grievances, and claims against physicians: does tort reform make a difference? J Healthc Risk Manag 30, 32–42. https://doi.org/10.1002/jhrm.20042

4. Alvarez Baranga, M.J., 2023. Right and duty of the doctor and the patient" Hospital mediation: reality or utopia? Louvain Medical 142, 275–287.
5. Ando, T., 2011. Healthcare mediation model for nerologists. Presented at the Clinical Neurology, pp. 827–829. https://doi.org/10.5692/clinicalneurol.51.827
6. Bergman, E.J., 2015. Identifying Sources of Clinical Conflict: A Tool for Practice and Training in Bioethics Mediation. J Clin Ethics 26, 315–323.
7. Bergman, E.J., 2013. A response to Dubler's commentary on "surmounting elusive barriers: the case for bioethics mediation". J Clin Ethics 24, 144–147.
8. Beyleveld, D., Brownsword, R., Wallace, S., 2002. Clinical ethics committees: clinician support or crisis management? HEC Forum 14, 13–25. https://doi.org/10.1023/a:1020965130205
9. Blanco Portillo, A., García-Caballero, R., Real de Asúa, D., Olaciregui Dague, K., Herreros, B., 2024. What Ethics Support for Resolving Ethical Conflicts Do Internists Use in Spanish Hospitals? J Bioeth Inq 21, 285–293. https://doi.org/10.1007/s11673-023-10276-1
10. Bowen, T., 2009. Using mediation in situations of withholding or withdrawing life-sustaining treatment: a New South Wales perspective.
11. Bowman, K.W., 2000. Communication, negotiation, and mediation: dealing with conflict in end-of-life decisions. J Palliat Care 16 Suppl, S17-23.
12. Brazg, T., Lindhorst, T., Dudzinski, D., Wilfond, B., 2016. \Defining Patient Advocacy for the Context of Clinical Ethics Consultation: A Review of the Literature and Recommendations for Consultants. J Clin Ethics 27, 176–184.
13. Brown, C.E., Marshall, A.R., Cueva, K.L., Snyder, C.R., Kross, E.K., Young, B.A., 2024. Physician Perspectives on Addressing Anti-Black Racism. JAMA Netw Open 7, e2352818. https://doi.org/10.1001/jamanetworkopen.2023.52818
14. Buchanan, S.F., Desrochers, J.M., Henry, D.B., Thomassen, G., Barrett, P.H.J., 2002. A mediation/medical advisory panel model for resolving disputes about end-of-life care. J Clin Ethics 13, 188–202.
15. Casarett, D.J., Daskal, F., Lantos, J., 1998. The authority of the clinical ethicist. Hastings Cent Rep 28, 6–11.
16. Chen, P.-Y., Fu, C.-P., Wang, C.-C., 2023. Narratives in the medicolegal field from the perspective of physicians involved in medical dispute mediation meetings in Taiwan. Heliyon 9, e13716. https://doi.org/10.1016/j.heliyon.2023.e13716
17. Cheng, K.K., le Roux-Kemp, A., 2017. Mediation and resolving disputes involving emergency nurses in Hong Kong: A legal empirical inquiry. Hong Kong Law Journal 47, 763–791.
18. Chiarchiaro, J., White, D.B., Ernecoff, N.C., Buddadhumaruk, P., Schuster, R.A., Arnold, R.M., 2016. Conflict Management Strategies in the ICU Differ Between Palliative Care Specialists and Intensivists. Crit Care Med 44, 934–942. https://doi.org/10.1097/CCM.0000000000001583
19. Cline, C., 2012. Why some conflicts involving "'difficult' patients" should remain outside the province of the ethics consultation service. Am J Bioeth 12, 16–18. https://doi.org/10.1080/15265161.2012.666166
20. Craig, K., 1993. Medical mediation. Med War 9, 62–65. https://doi.org/10.1080/07488009308409079
21. Craig, Y.J., 1996. Patient decision-making: medical ethics and mediation. J Med Ethics 22, 164–167. https://doi.org/10.1136/jme.22.3.164

22. Crigger, B.J., 1995. Negotiating the moral order: paradoxes of ethics consultation. Kennedy Inst Ethics J 5, 89–112. https://doi.org/10.1353/ken.0.0066
23. DeAngelo, L.M., 2000. Mediation in health care settings: Some theoretical and practical concepts. Journal of Clinical Psychology in Medical Settings 7, 133–139.
24. Decoulx, M., Scherpereel, P., 2013. [For a more humane hospital: experience of medical mediators]. Presse Med 42, 389–394. https://doi.org/10.1016/j.lpm.2012.06.009
25. Díaz Rivera, Y.A., 2015. [Project for the Creation of a Medical or Hospital Ethical Committee at a Local Level in the San Miguel Arcangel Hospital, District of San Miguelito, Province of Panama. Year 2013]. Cuad Bioet 26, 292–301.
26. Dimitrov, K., Miteva-Katrandzhieva, T., 2024. Mediation in Healthcare: Enhancing Conflict Resolution Between Patients and Physicians Beyond the Courtroom. Cureus 16, e75487. https://doi.org/10.7759/cureus.75487
27. Dimitrov, K.Y., Miteva-Katrandzhieva, T., 2025. Exploring Patient Awareness and the Feasibility of Mediation in Healthcare: A Pilot Study in Bulgaria. Healthcare 13, 629. https://doi.org/10.3390/healthcare13060629
28. Dubler, N.N., 2013a. The art of the chart note in clinical ethics consultation and bioethics mediation: conveying information that can be understood and evaluated. J Clin Ethics 24, 148–155.
29. Dubler, N.N., 2013b. Commentary on Bergman: "yes... but". J Clin Ethics 24, 25–31.
30. Dubler, N.N., 2011. Commentary on Fiester's "Ill-placed democracy: ethics consultations and the moral status of voting". J Clin Ethics 22, 373–379.
31. Dubler, N.N., 2002. Mediating disputes in managed care: resolving conflicts over covered services. J Health Care Law Policy 5, 479–501.
32. Dubler, N.N., 1998. Mediation and managed care. J Am Geriatr Soc 46, 359–364. https://doi.org/10.1111/j.1532-5415.1998.tb01054.x
33. DuBois, J.M., 2024. The Bioethicist as Healer. Hastings Cent Rep 54, 2. https://doi.org/10.1002/hast.4901
34. DuVal, G., Sartorius, L., Clarridge, B., Gensler, G., Danis, M., 2001. What triggers requests for ethics consultations? J Med Ethics 27 Suppl 1, i24-29. https://doi.org/10.1136/jme.27.suppl_1.i24
35. Eves, M.M., Esplin, B.S., 2015. "She Just Doesn't Know Him Like We Do": Illuminating Complexities in Surrogate Decision Making. J Clin Ethics 26, 350–354.
36. Felder, R.M., 2024. Toward a new clinical pragmatism: method in clinical ethics consultation. Med Health Care Philos 27, 445–454. https://doi.org/10.1007/s11019-024-10219-6
37. Fiester, A., 2025a. Defending Dubler's Legacy: Relocating the Role of Conflict Management from the Ethics Consultation Service to Patient and Guest Relations. The Journal of Clinical Ethics 36, 385–395. https://doi.org/10.1086/737393
38. Fiester, A., 2025b. The Transformative Power of Reasons Relitigates Concerns about Non-Facilitated Healthcare Ethics Consultation. The American Journal of Bioethics 25, 24–26. https://doi.org/10.1080/15265161.2025.2554776

39. Fiester, A., 2024. The “Ladder of Inference” as a Conflict Management Tool: Working with the “Difficult” Patient or Family in Healthcare Ethics Consultations. HEC Forum 36, 31–44. https://doi.org/10.1007/s10730-022-09476-w
40. Fiester, A., 2015. Neglected ends: clinical ethics consultation and the prospects for closure. Am J Bioeth 15, 29–36. https://doi.org/10.1080/15265161.2014.974770
41. Fiester, A., 2013. A dubious export: the moral perils of American-style ethics consultation. Bioethics 27, ii–iii. https://doi.org/10.1111/bioe.12011
42. Fiester, A., 2012. Mediation and advocacy. Am J Bioeth 12, 10–11. https://doi.org/10.1080/15265161.2012.692442
43. Fiester, A., 2007a. Mediation and moral aporia. J Clin Ethics 18, 355–356.
44. Fiester, A., 2007b. The failure of the consult model: why “mediation” should replace “consultation”. Am J Bioeth 7, 31–32. https://doi.org/10.1080/15265160601109234
45. Fiester, A.M., 2011. Ill-Placed Democracy: Ethics Consultations and the Moral Status of Voting. The Journal of Clinical Ethics 22, 363–372. https://doi.org/10.1086/JCE201122409
46. Flicker, L., Powell, T., 2025. The House That Nancy Built. The Journal of Clinical Ethics 36, 309–314. https://doi.org/10.1086/737387
47. Forbat, L., Barclay, S., 2019. Reducing healthcare conflict: outcomes from using the conflict management framework. Arch Dis Child 104, 328–332. https://doi.org/10.1136/archdischild-2018-315647
48. Forbat, L., Simons, J., Sayer, C., Davies, M., Barclay, S., 2017. Training paediatric healthcare staff in recognising, understanding and managing conflict with patients and families: findings from a survey on immediate and 6-month impact. Arch Dis Child 102, 250–254. https://doi.org/10.1136/archdischild-2016-310737
49. Forbat, L., Teuten, B., Barclay, S., 2015. Conflict escalation in paediatric services: findings from a qualitative study. Arch Dis Child 100, 769–773. https://doi.org/10.1136/archdischild-2014-307780
50. Fournier, V., Spranzi, M., Foureur, N., Brunet, L., 2015. The “Commitment Model” for Clinical Ethics Consultations: Society’s Involvement in the Solution of Individual Cases. J Clin Ethics 26, 286–296.
51. François, K., Lobb, E., Barclay, S., Forbat, L., 2017. The nature of conflict in palliative care: A qualitative exploration of the experiences of staff and family members. Patient Educ Couns 100, 1459–1465. https://doi.org/10.1016/j.pec.2017.02.019
52. Gallagher, T.H., Mello, M.M., Sage, W.M., Bell, S.K., McDonald, T.B., Thomas, E.J., 2018. Can Communication-And-Resolution Programs Achieve Their Potential? Five Key Questions. Health Aff (Millwood) 37, 1845–1852. https://doi.org/10.1377/hlthaff.2018.0727
53. Gatter, R., 2004. Institutionally sponsored mediation and the emerging medical trust movement in the U.S. Med Law 23, 201–210.
54. Gatter, R., 1999. Unnecessary adversaries at the end of life: mediating end-of-life treatment disputes to prevent erosion of physician-patient relationships. Boston Univ Law Rev 79, 1091–1137.
55. Geppert, C.M.A., Shelton, W., 2016. Health Care Ethics Committees as Mediators of Social Values and the Culture of Medicine. AMA J Ethics 18, 534–539. https://doi.org/10.1001/journalofethics.2016.18.5.msoc1-1605

56. Gibson, J.M., 1994. Mediation for ethics committees: a promising process. Generations 18, 58–60.
57. Gibson, K., 1999. Mediation in the medical field. Is neutral intervention possible? Hastings Cent Rep 29, 6–13.
58. Glover, A., Bertino, J., 2018. Bioethics Mediation: A Practical Approach to Physician Assistant Ethics Education. J Physician Assist Educ 29, 247–250. https://doi.org/10.1097/JPA.0000000000000222
59. Goranova-Spasova, R., Gradinarova, N., 2022. APPLICATION OF MEDIATION IN THE FIELD OF HEALTHCARE. General Medicine 24, 36–40.
60. Greco, E., 2025. Disputing Death: Medical Futility Laws and Procedures to Facilitate End of Life Discussions among Patients, Family, and Practitioners. SDL Rev. 70, 130.
61. Hauschildt, K., De Vries, R., 2020. Reinforcing medical authority: clinical ethics consultation and the resolution of conflicts in treatment decisions. Sociol Health Illn 42, 307–326. https://doi.org/10.1111/1467-9566.13003
62. Herron, P.D., 2025. Honoring Chosen Family: Revisiting the Doctor-Proxy Relationship. The Journal of Clinical Ethics 36, 332–338. https://doi.org/10.1086/737391
63. Hoffman, D.N., Strand, G.R., 2024. "Sit down and thrash it out": opportunities for expanding ethics consultation during conflict resolution in long-term care. New Bioeth 30, 152–162. https://doi.org/10.1080/20502877.2024.2330275
64. Howe, E.G., 2025. Nancy Dubler's Contributions to Clinical Ethics Consultation. The Journal of Clinical Ethics 36, 295–302. https://doi.org/10.1086/737396
65. Hyman, C.S., Liebman, C.B., Schechter, C.B., Sage, W.M., 2010. Interest-based mediation of medical malpractice lawsuits: a route to improved patient safety? J Health Polit Policy Law 35, 797–828. https://doi.org/10.1215/03616878-2010-028
66. Johal, H.K., Birchley, G., Huxtable, R., 2022. Exploring physician approaches to conflict resolution in end-of-life decisions in the adult intensive care unit: protocol for a systematic review of qualitative research. BMJ Open 12, e057387. https://doi.org/10.1136/bmjopen-2021-057387
67. Kass, J.S., Rose, R.V., 2016. Medical Malpractice Reform--Historical Approaches, Alternative Models, and Communication and Resolution Programs. AMA J Ethics 18, 299–310. https://doi.org/10.1001/journalofethics.2016.18.3.pfor6-1603
68. Kayser, J.B., 2015. Mediation Training for the Physician: Expanding the Communication Toolkit to Manage Conflict. J Clin Ethics 26, 339–341.
69. Kayser, J.B., Kaplan, L.J., 2020. Conflict Management in the ICU. Crit Care Med 48, 1349–1357. https://doi.org/10.1097/CCM.0000000000004440
70. Khushf, G., 2019. When Religious Language Blocks Discussion About Health Care Decision Making. HEC Forum 31, 151–166. https://doi.org/10.1007/s10730-019-09371-x
71. Kolak, J., Hulkower, A., 2025. Dialogic Engagement and the Epistemic Norms of Bioethics Mediation. The Journal of Clinical Ethics 36, 339–352. https://doi.org/10.1086/737395
72. Kwon, O.-T., Seon, J.G., Kim, S.Y., 2012. Discussions and implications of the recent enactment & revision of the healthcare law. J Korean Med Sci 27 Suppl, S82-87. https://doi.org/10.3346/jkms.2012.27.S.S82
73. Latham, S., 2015. Facilitated discussion: good and good for you. Am J Bioeth 15, 58–59. https://doi.org/10.1080/15265161.2015.978625

74. Lee, D.W.H., Lai, P.B.S., 2015. The practice of mediation to resolve clinical, bioethical, and medical malpractice disputes. Hong Kong Med J 21, 560–564. https://doi.org/10.12809/hkmj154615
75. Lindsey, J., Doyle, M., Wazynska-Finck, K., 2025a. Securing therapeutic justice through mediation: the challenge of medical treatment disputes. Legal stud. 45, 40–57. https://doi.org/10.1017/lst.2024.39
76. Lindsey, J., Francis, G., Doyle, M., 2025b. Mediation of medical treatment disputes: a therapeutic justice model end of project report.
77. Linney, M., Hain, R.D.W., Wilkinson, D., Fortune, P.-M., Barclay, S., Larcher, V., Fitzgerald, J., Arkell, E., 2019. Achieving consensus advice for paediatricians and other health professionals: on prevention, recognition and management of conflict in paediatric practice. Arch Dis Child 104, 413–416. https://doi.org/10.1136/archdischild-2018-316485
78. Lyons, O., Forbat, L., Menson, E., Chisholm, J.C., Pryde, K., Conlin, S., Felton, V., Ingle, S., McKenzie, C., Ramachandran, R., Sayer, C., Snowball, C., Strachan-Gadsby, E., Tisovszky, N., Barclay, S., 2021. Transforming training into practice with the conflict management framework: a mixed methods study. BMJ Paediatr Open 5, e001088. https://doi.org/10.1136/bmjpo-2021-001088
79. Madden, S., Martin, D.K., Downey, S., Singer, P.A., 2005. Hospital priority setting with an appeals process: a qualitative case study and evaluation. Health Policy 73, 10–20. https://doi.org/10.1016/j.healthpol.2004.11.002
80. Martin, P.A., 1999. Bioethics and the whole: pluralism, consensus, and the transmutation of bioethical methods into gold. J Law Med Ethics 27, 316–327, 294. https://doi.org/10.1111/j.1748-720x.1999.tb01466.x
81. Martínez-López, J.Á., Lozano, E.B., Gómez, P.M., Ayala, J.A.G., 2023. THE RIGHT TO EUTHANASIA: MEDIATION IN END-OF-LIFE DECISION-MAKING. Prisma Social 43, 262–282.
82. Matchett, N.J., 2015. Philosophical counseling as an alternative process to bioethics mediation. Am J Bioeth 15, 56–58. https://doi.org/10.1080/15265161.2014.974774
83. Maung, A.A., Toevs, C.C., Kayser, J.B., Kaplan, L.J., 2015. Conflict management teams in the intensive care unit: A concise definitive review. J Trauma Acute Care Surg 79, 314–320. https://doi.org/10.1097/TA.0000000000000728
84. McClimans, L., Pressgrove, G., Campbell, E., 2019. Objectives and outcomes of clinical ethics services: a Delphi study. J Med Ethics 45, 761–769. https://doi.org/10.1136/medethics-2018-105203
85. McGreevy, J., 2015. In the Ethos of the Safety Net: An Expanded Role for Clinical Ethics Mediation. J Clin Ethics 26, 336–338.
86. Meller, S., Barclay, S., 2011. Mediation: an approach to intractable disputes between parents and paediatricians. Arch Dis Child 96, 619–621. https://doi.org/10.1136/adc.2010.191833
87. Miles, F., Barclay, S., Menson, E., Shepherd, T., Webster, L., 2023. Boldly going… Introducing conflict management training to Starship Children's Hospital. J Paediatr Child Health 59, 424–426. https://doi.org/10.1111/jpc.16347
88. Miller, R.B., 2002. Extramural ethics consultation: reflections [correction of relections] on the mediation/medical advisory panel model and a further proposal. J Clin Ethics 13, 203–215.

89. Mitello, L., 2004. [When the patient asks for counselling, when the patient doesn't ask for counselling, when the patient refuses therapy]. Prof Inferm 57, 216–221.
90. Morreim, E.H., 2025. A Great Gift … with Great Caveats: Bioethics Mediation versus Bona Fide Mediation. The Journal of Clinical Ethics 36, 375–384. https://doi.org/10.1086/737397
91. Morreim, H., 2024. From Philosopher in Residence to Healthcare Mediation. J. Law. Med. Ethics 52, 321–323. https://doi.org/10.1017/jme.2024.96
92. Morreim, H., 2015. Conflict Resolution in the Clinical Setting: A Story Beyond Bioethics Mediation. J Law Med Ethics 43, 843–856. https://doi.org/10.1111/jlme.12324
93. Munuera Gómez, P., 2020. [Medical mediation in the international context]. Rev Med Chil 148, 792–798. https://doi.org/10.4067/S0034-98872020000600792
94. Nakanishi, T., 2014. Disclosing unavoidable causes of adverse events improves patients' feelings towards doctors. Tohoku J Exp Med 234, 161–168. https://doi.org/10.1620/tjem.234.161
95. Nelson, C.M., Nazareth, B.A., 2013. Nonbeneficial treatment and conflict resolution: building consensus. Perm J 17, 23–27. https://doi.org/10.7812/TPP/12-124
96. Nicolas, P., Sullivan, L.S., Chuang, E., 2025. Mediation of Medical Distrust due to Racial Injustice: The Legacy of Nancy Dubler. The Journal of Clinical Ethics 36, 363–374. https://doi.org/10.1086/737394
97. Oh, E.H., Shin, J.E., Bae, J.Y., Lee, Y.S., Park, Y., Kwon, Y.H., Paik, C.N., Lee, J.K., Lee, T.H., 2025. Medical disputes involving lower gastrointestinal endoscopies: cases from the Korean Medical Dispute Mediation and Arbitration Agency. Korean J Intern Med 40, 404–426. https://doi.org/10.3904/kjim.2024.343
98. Omelianchuk, A., Ansari, A.A., Parsi, K., 2024. What Is It That You Want Me To Do? Guidance for Ethics Consultants in Complex Discharge Cases. HEC Forum 36, 513–526. https://doi.org/10.1007/s10730-023-09517-y
99. Ong, C., 2013. Medical mediation: bringing everyone to the table. Bull Am Coll Surg 98, 17–20.
100. Orr, R.D., 2002. Working toward peace in the clinical setting: the role of clinical ethics in conflict resolution. Todays Christ Dr 33, 26–30.
101. Orr, R.D., 2001. Methods of conflict resolution at the bedside. Am J Bioeth 1, 45–46. https://doi.org/10.1162/152651601317139397
102. Orr, R.D., deLeon, D.M., 2000. The role of the clinical ethicist in conflict resolution. J Clin Ethics 11, 21–30.
103. Orr, R.D., Marshall, P.A., Osborn, J., 1995. Cross-cultural considerations in clinical ethics consultations. Arch Fam Med 4, 159–164. https://doi.org/10.1001/archfami.4.2.159
104. Paquette, E.T., Kolaitis, I.N., 2015. Intensive Care, Intense Conflict: A Balanced Approach. J Clin Ethics 26, 346–349.
105. Parker, M.J., 2024. Bioethics and the value of disagreement. J Med Ethics jme-2024-110174. https://doi.org/10.1136/jme-2024-110174
106. Pavlish, C., Brown-Saltzman, K., Fine, A., Jakel, P., 2013. Making the call: a proactive ethics framework. HEC Forum 25, 269–283. https://doi.org/10.1007/s10730-013-9213-5

107. Perangin-Angin, T.A., Lamria Sintia Silaban, Sonya Airini Batubara, Jusnizar Sinaga, 2025. Mediation as an Alternative to Legal Dispute Resolution in Health Services in Hospitals. JS 11, 192–202. https://doi.org/10.33506/js.v11i1.3898
108. Phillipson, J., Barclay, S., Menson, E., Lyons, O., 2024. Healthcare decision-makers' perspectives on evaluating conflict management training in paediatric healthcare: a utilisation-focused qualitative study. BMJ Paediatr Open 8. https://doi.org/10.1136/bmjpo-2024-003047
109. Phua, J., 2022. Healthcare Mediation: Bridging the Gap, in: Contemporary Issues in Mediation: Volume 7. pp. 45–52. https://doi.org/10.1142/9789811268724_0005
110. Powell, T., Hulkower, A., 2017. A Good Death. Hastings Cent Rep 47, 28–29. https://doi.org/10.1002/hast.669
111. Przylepa-Lewak, A., 2023. Contemporary challenges of medical mediation. Krytyka Prawa 15, 238–254. https://doi.org/10.7206/kp.2080-1084.631
112. Rangkutir, R., Risdawati, I., 2024. THE ROLE OF HOSPITAL MANAGEMENT IN RESOLUTION OF MEDICAL DISPUTES THROUGH MEDIATION PATHS IN THE HOSPITAL. Presented at the International Conference on Health Science, Green Economics, Educational Review and Technology, pp. 12–24.
113. Regis, C., Poitras, J., 2010. Healthcare mediation and the need for apologies. Health law journal 18, 31–49.
114. Reynolds, D.F., 1994. Consultectonics: ethics committee case consultation as mediation. Bioethics Forum 10, 54–60.
115. Ribeiro, W.C., 2018. Mediation as a means of resolving conflicts in the healthcare area. Revista de Direito Sanitario 18, 62–75. https://doi.org/10.11606/issn.2316-9044.v18i3p62-76
116. Rotily, M., Lamouroux-Delay, A., Cristina Rojas-Vergara, A., 2025. Recours à la médiation en santé chez les patients en situation de précarité : l'exemple du recours aux urgences: Santé Publique 37, 217–228. https://doi.org/10.3917/spub.255.0217
117. Saito, Y., Takeda, K., Akama, N., Yamauchi, T., 2012. Significance of training in healthcare mediation to resolve conflicts between health professionals and patients. Presented at the IRYO - Japanese Journal of National Medical Services, pp. 566–569.
118. Sari, I., 2025. Alternative Dispute Resolution In Medical Dispute Resolution: Initiating The Establishment Of An Alternative Medical Dispute Resolution Institution In Indonesia. Fox Justi: Jurnal Ilmu Hukum 15, 392–408.
119. Savage, T.A., Parson, J., Zollman, F., Kirschner, K.L., 2009. Rehabilitation team disagreement: guidelines for resolution. PM R 1, 1091–1097. https://doi.org/10.1016/j.pmrj.2009.09.017
120. Schildmann, J., Nadolny, S., Haltaufderheide, J., Gysels, M., Vollmann, J., Bausewein, C., 2019. Do we understand the intervention? What complex intervention research can teach us for the evaluation of clinical ethics support services (CESS). BMC Med Ethics 20, 48. https://doi.org/10.1186/s12910-019-0381-y
121. Schlairet, M.C., 2009. Bioethics mediation: the role and importance of nursing advocacy. Nurs Outlook 57, 185–193. https://doi.org/10.1016/j.outlook.2008.10.006
122. Scofield, G.R., 1995. In medias res: the ethicist as mediator. Trends Health Care Law Ethics 10, 7–14.

123. Selandari, J.O., de la Portilla, M., Ciruzzi, M.S., Couceiro, C., García, H.O., Iervolino, M. de L.Á., Marín, D.N., Miranda, C., Novali, L., Ortega, L., Ponce, C., Puccar, P., Quintana, S.G., Rodríguez, E., Ledesma, F., 2022. Feasibility, effectiveness, and satisfaction achieved by the transdisciplinary intervention of a clinical-hospital ethics committee. A qualitative and quantitative study. Arch Argent Pediatr 120, 30–38. https://doi.org/10.5546/aap.2022.eng.30

124. Shen, Y., Li, G., Tang, Z., Wang, Q., Zhang, Z., Hao, X., Han, X., 2024. Analysis of the characteristics, efficiency, and influencing factors of third-party mediation mechanisms for resolving medical disputes in public hospitals in China. BMC Public Health 24, 1823. https://doi.org/10.1186/s12889-024-19366-0

125. Singer, P.A., Barker, G., Bowman, K.W., Harrison, C., Kernerman, P., Kopelow, J., Lazar, N., Weijer, C., Workman, S., 2001. Hospital policy on appropriate use of life-sustaining treatment. University of Toronto Joint Centre for Bioethics/Critical Care Medicine Program Task Force. Crit Care Med 29, 187–191. https://doi.org/10.1097/00003246-200101000-00037

126. Sproule, R., 1989. Update on the medical mediation panels. Wis Med J 88, 25–26.

127. Sriwidodo, J., Wahid, S.H., Kususiyanah, A., 2025. Toward Equitable Healthcare: A Medical Dispute Resolution Framework to Address Medical Supply Delays in Health Law. J. Leg. Aff. Dispute Resolut. Eng. Constr. 17, 04525040. https://doi.org/10.1061/JLADAH.LADR-1298

128. Stevenson, J., Clinch, A., Ftanou, M., Delany, C., 2024. What is known about the role of clinical ethics services in cancer care? A systematic/narrative literature review. BMJ Support Palliat Care 14, 403–410. https://doi.org/10.1136/spcare-2023-004300

129. Tan, H.S., 2025. What's Best and Who Decides for Seriously Ill Infants? A Malaysian Perspective. Asian Bioeth Rev 17, 343–355. https://doi.org/10.1007/s41649-024-00339-9

130. Tantiono, P., Darma, I.M.W., Kurniawan, I.G.A., 2025. The Rights of Families of Terminal Patients to Refuse Futile Treatment: Legal and Ethical Limitations. J.Pena.Justisia 24, 5886–5908. https://doi.org/10.31941/pj.v24i2.7104

131. Teremetskyi, V., Tokarieva, K., Batryn, O., Myrza, S., Mosondz, S., Matviichuk, A., 2024. Mediation as an Effective Mechanism for Resolving Disputes Caused by Medical Errors.

132. The Healthcare Conflict Scale: development, validation and reliability testing of a tool for use across clinical settings., 2019. . England. https://doi.org/10.1080/13561820.2019.1593117

133. Trotter, G., 2002. Bioethics and healthcare reform: a Whig response to weak consensus. Camb Q Healthc Ethics 11, 37–51. https://doi.org/10.1017/s096318010210106x

134. Turner, K., 2024. Hidden Fault Lines in the Bedrock: A Critical Examination of Surrogate Decision-Making Standards in Ethics Consultation. J Clin Ethics 35, 155–168. https://doi.org/10.1086/730875

135. Voultsos, P., Tsompanian, A., Tsaroucha, A.K., 2021. The medical futility experience of nursing professionals in Greece. BMC Nurs 20, 254. https://doi.org/10.1186/s12912-021-00785-y

136. Wada, Y., 2012. Suggestions from sites of healthcare mediation. Presented at the IRYO - Japanese Journal of National Medical Services, pp. 553–555.
137. Waldman, E., 2003. Mediating difference: normative conflict as opportunity. Am J Bioeth 3, 25–27. https://doi.org/10.1162/152651603766436162
138. Walton, M.K., 2015. Patient-Centered Care and the Mediator's Skills. J Clin Ethics 26, 333–335.
139. Wang, M., Liu, G.G., Zhao, H., Butt, T., Yang, M., Cui, Y., 2020. The role of mediation in solving medical disputes in China. BMC Health Serv Res 20, 225. https://doi.org/10.1186/s12913-020-5044-7
140. Watkins, L.T., Sacajiu, G., Karasz, A., 2007. The role of the bioethicist in family meetings about end of life care. Soc Sci Med 65, 2328–2341. https://doi.org/10.1016/j.socscimed.2007.06.025
141. Weaver, M.S., Boss, R.D., Christopher, M.J., Gray, T.F., Harman, S., Madrigal, V.N., Michelson, K.N., Paquette, E.T., Pentz, R.D., Scarlet, S., Ulrich, C.M., Walter, J.K., 2022. Top Ten Tips Palliative Care Clinicians Should Know About Their Work's Intersection with Clinical Ethics. J Palliat Med 25, 656–661. https://doi.org/10.1089/jpm.2021.0521
142. Weinstein, M.S., 2015. A Second Opinion: A Case Narrative on Clinical Ethics Mediation. J Clin Ethics 26, 331–332.
143. Welie, J.V., 1998. Clinical ethics: theory or practice? Theor Med Bioeth 19, 295–312. https://doi.org/10.1023/a:1009974201726
144. West, M.B., Gibson, J.M., 1992. Facilitating medical ethics case review: what ethics committees can learn from mediation and facilitation techniques. Camb Q Healthc Ethics 1, 63–74. https://doi.org/10.1017/s0963180100000098
145. Widjaja, G., 2025. SETTLEMENT OF MEDICAL DISPUTES DUE TO MINOR OFFENCES BY HEALTH WORKERS THROUGH MEDIATION. INJOSEDU: International Journal of Social and Education 2, 1846–1855.
146. Wilkinson, D., Barclay, S., Savulescu, J., 2018. Disagreement, mediation, arbitration: resolving disputes about medical treatment. Lancet 391, 2302–2305. https://doi.org/10.1016/S0140-6736(18)31220-0
147. Wood, E., Karp, N., 1994. Mediation: reframing care conflicts in nursing homes. Generations 18, 54–57.
148. Wright, L., Ross, K., Daar, A.S., 2005. The roles of a bioethicist on an organ transplantation service. Am J Transplant 5, 821–826. https://doi.org/10.1046/j.1600-6143.2005.00764.x
149. Yoo, S.H., Kim, Y., Choi, W., Shin, J., Kim, M.S., Park, H.Y., Keam, B., Yim, J.-J., 2023. Ethical Issues Referred to Clinical Ethics Support at a University Hospital in Korea: Three-Year Experience After Enforcement of Life-Sustaining Treatment Decisions Act. J Korean Med Sci 38, e182. https://doi.org/10.3346/jkms.2023.38.e182
150. Zákány, J., 2025. ALTERNATIVE DISPUTE RESOLUTION IN HEALTHCARE SECTOR IN HUNGARY: THE ROLE OF CONCILIATION BOARDS. Curentul Juridic 102, 61–72.
151. Zanni, A., 2014. Organ transplantation in Greece: the need for mediation. Transplant Proc 46, 3164–3167. https://doi.org/10.1016/j.transproceed.2014.09.157

152. Zhang, J., 2025. Reevaluating Benevolent Deception: A Trust-Oriented Approach to Ethical Mediation in Multicultural Healthcare. The Journal of Clinical Ethics 36, 353–362. https://doi.org/10.1086/737398
